# Proteomic analysis identifies plasma correlates of remote ischemic conditioning in the context of experimental traumatic brain injury

**DOI:** 10.1038/s41598-020-69865-4

**Published:** 2020-07-31

**Authors:** Maha Saber, Khyati V. Pathak, Marissa McGilvrey, Krystine Garcia-Mansfield, Jordan L. Harrison, Rachel K. Rowe, Jonathan Lifshitz, Patrick Pirrotte

**Affiliations:** 1 BARROW Neurological Institute at Phoenix Children’s Hospital, Phoenix, AZ USA; 2 Child Health, University of Arizona College of Medicine-Phoenix, 425 N 5th street ABC1, Phoenix, AZ USA; 3 Collaborative Center for Translational Mass Spectrometry, Translational Genomics Research Institute, Phoenix, AZ USA; 4 Phoenix VA Health Care System, Phoenix, AZ USA

**Keywords:** Biomarkers, Neuroscience

## Abstract

Remote ischemic conditioning (RIC), transient restriction and recirculation of blood flow to a limb after traumatic brain injury (TBI), can modify levels of pathology-associated circulating protein. This study sought to identify TBI-induced molecular alterations in plasma and whether RIC would modulate protein and metabolite levels at 24 h after diffuse TBI. Adult male C57BL/6 mice received diffuse TBI by midline fluid percussion or were sham-injured. Mice were assigned to treatment groups 1 h after recovery of righting reflex: sham, TBI, sham RIC, TBI RIC. Nine plasma metabolites were significantly lower post-TBI (six amino acids, two acylcarnitines, one carnosine). RIC intervention returned metabolites to sham levels. Using proteomics analysis, twenty-four putative protein markers for TBI and RIC were identified. After application of Benjamini–Hochberg correction, actin, alpha 1, skeletal muscle (ACTA1) was found to be significantly increased in TBI compared to both sham groups and TBI RIC. Thus, identified metabolites and proteins provide potential biomarkers for TBI and therapeutic RIC in order to monitor disease progression and therapeutic efficacy.

## Introduction

Traumatic brain injury (TBI) is a nondiscriminatory event that can affect any person at any age with any medical background. Approximately 69 million people sustain a TBI annually worldwide^1^. In the United States alone, 2.87 million people suffer from TBI each year, and from those, approximately 288,000 patients are hospitalized, 56,800 people die, and over 90,000 live with permanent disabilities (CDC). Those that survive the initial insult can suffer from chronic cognitive issues and neurodegenerative diseases. A single episode of TBI can trigger progressive neurodegenerative pathologies years after the initial insult^2–7^. TBI-induced neurodegenerative diseases often are diagnosed several years after the initial injury with few, if any, treatment options available to slow down or stop the progression of the disease^8^. The majority of TBI incidents go unreported or are undiagnosed due to unreliable or non-existent biological indicators. Recently approved blood biomarkers for TBI indicate the need for or support the use of computed tomography (CT) imaging for diagnosis, rather than monitoring disease progression or recovery^9^. Clinical care of a positive TBI diagnosis necessitates reliable monitoring of biomarkers to inform and guide treatment. Due to the absence of a monitoring biomarker, standard clinical protocols allow patients to return to action or duty based on unreliable self-reported symptom surveys^10^. Premature return to normal or strenuous activities can worsen the condition and induce chronic health-related issues, further exacerbating the risk for neurodegenerative diseases^8,11^.

Monitoring biomarkers can track the effectiveness of interventions on TBI-related symptoms and pathologies. Currently, biomarkers for TBI include S100 calcium-binding protein B (S-100B), neuron-specific enolase (NSE), ubiquitin C-terminal hydrolase isozyme L1 (UCH-L1) and glial fibrillary acidic protein (GFAP); expression are elevated in serum over 24 h after severe or suspected TBI in patients^12–14^. However, serial sampling of these proteins in serum revealed different temporal trajectories in patients with severe TBI, making them poor candidates to monitor TBI recovery^15^. Ideal monitoring biomarkers should match or predict the trajectory of symptoms and pathology, and further track a return to baseline of both the symptoms and level of biomarker with treatment efficacy. An additional limitation of NSE as a clinical biomarker is the possibility of sample contamination from artifactual hemolysis as a result of collecting or processing blood^16^. Though these biomarkers may be helpful for diagnostic measures, these biomarkers are not ideal to monitor disease progression or the effect of treatment.

Though S-100B and NSE are not ideal candidates for monitoring biomarkers, they are consistently elevated after severe clinical brain injury. Remote ischemic conditioning (RIC) reduced serum levels of S-100B and NSE 24 h after a severe brain injury when RIC was administered 1 h after hospital admission^17^. RIC is the transient and repeated restriction of blood flow to a limb or non-vital organ. The procedure is cost effective, easy to teach, accessible, and carries very little risk, as the procedure only requires a tourniquet or similar method to briefly reduce blood supply to the limb. RIC has been employed clinically to reduce ischemic damage during myocardial infarctions and stroke^17–19^. As an intervention for severe TBI, RIC reduced levels of NSE and S-100B in patient sera at 6- and 24-h post-RIC treatment at time of admission^20^. Although several mechanisms may explain RIC efficacy, the molecular underpinnings of RIC have not been substantiated. Untargeted biomarker discovery can uncover mechanisms of RIC following acute neurological injury. We hypothesized that RIC would reverse TBI-induced molecular alterations, some of which may serve as biomarkers to monitor recovery.

This study profiled the plasma proteome and metabolome at 24 h after a mouse model of diffuse TBI and subsequent RIC intervention. Midline fluid percussion injury (mFPI) was selected to model a clinically relevant diffuse TBI and is one of the best characterized models for concussive-like pathologies and behaviors^21–23^. Given the remote application of RIC and potential for blood-borne therapeutic molecules, circulating metabolome and proteome was used to determine significantly differentiated metabolites and proteins between RIC-treated and TBI groups. Targeted metabolomic analysis discerned 94 differentially abundant metabolites, nine of which were significantly lower in TBI groups and returned to uninjured sham levels following RIC treatment. Proteomic analysis identified 24 potential protein biomarkers. This study identified potential new monitoring biomarkers for TBI-pathophysiology and RIC treatment.

## Results

### Diffuse TBI suppressed the mouse righting reflex time

**Figure 1:**
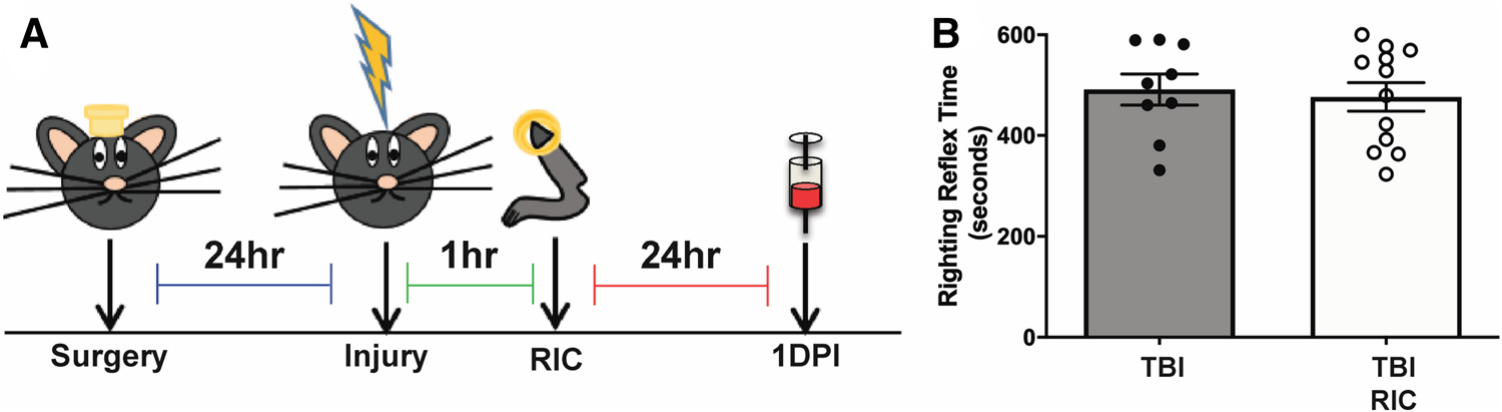
Study design and righting reflex times. (**A**) Study schematic. (**B**) Righting reflex times are shown in seconds. There were no significant differences in righting reflex times in either group. Uninjured sham mice had a righting reflex of less than 20 s (not shown).

In this study, RIC treatment in male mice was investigated following diffuse TBI by mFPI (Fig. 1A). Diffuse TBI suppressed the reflex for a mouse to correct their body position from the supine position (righting reflex). Shams included in the study had less than 20 s for their righting reflex times and were not included in the analysis. No significant difference in the righting reflex times was observed between TBI and TBI RIC (t(19) = 0.3477, p = 0.7319; Fig. 1B).

### TBI reduced plasma concentration of amino acids and RIC treatment restored the levels

**Figure 2:**
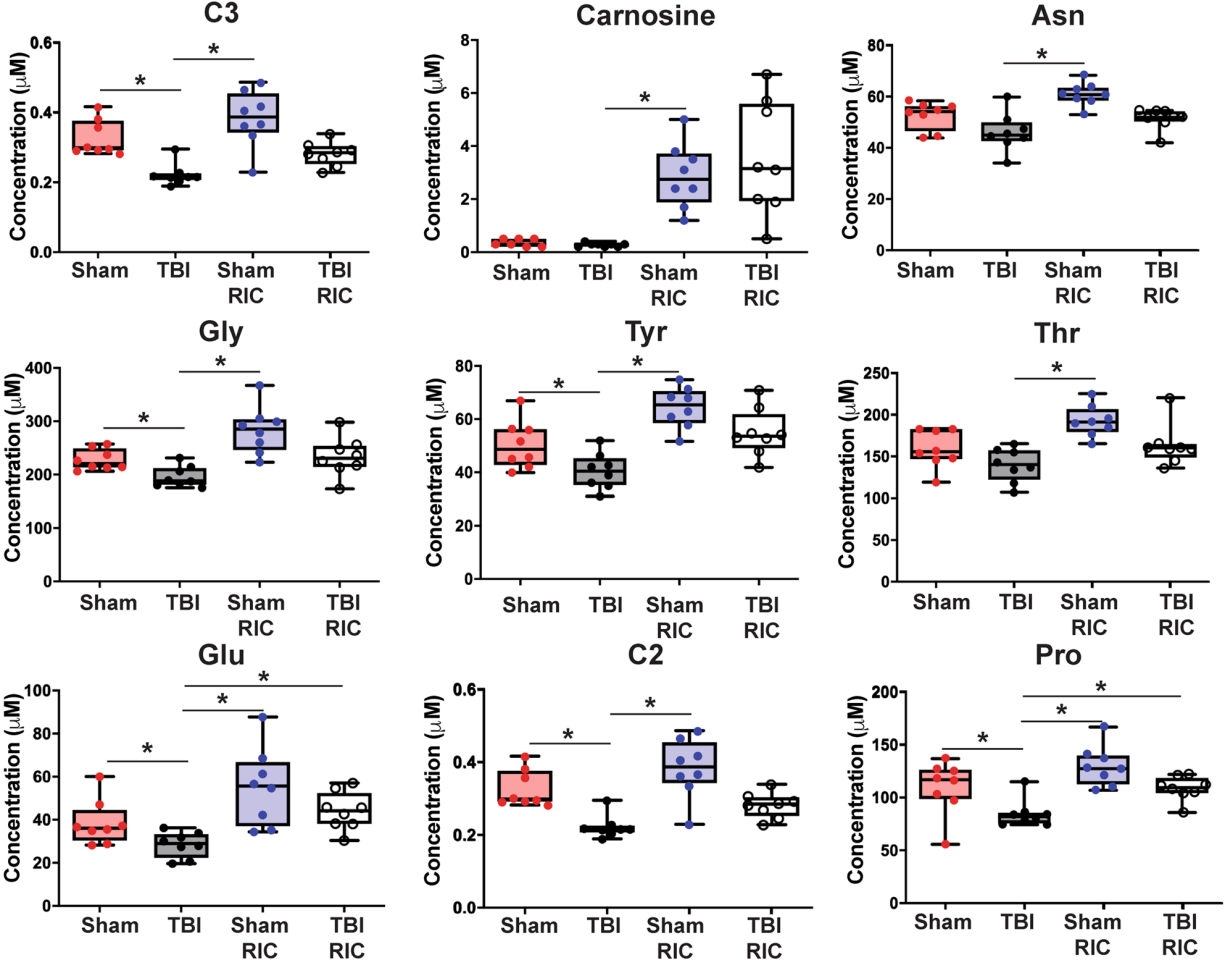
Box-Whisker plots representing concentrations of significantly different metabolites between sham, TBI, sham RIC and TBI RIC mice. The targeted metabolomics analysis was performed on plasma obtained from mice subjected to sham, TBI, sham RIC and TBI RIC treatments. The concentrations of metabolites were measured in µM unit. Significantly different metabolites (q-value < 0.05) across these groups were determined using Kruskal–Wallis test with Benjamini–Hochberg correction for multiple testing. All nine metabolites were statistically significant (q < 0.05): six amino acids (Asn, Pro, Glu, Gly, Thr and Tyr), carnosine, and two acylcarnitines (C2 and C3).

Targeted quantitative metabolomics analyses were performed for 185 metabolites using mass spectrometry. These included amino acids, biogenic amines, glycerophospholipids, sphingolipids, acylcarnitines, and hexose metabolites. The micromolar concentrations of 94 (50.8%) metabolites (14 amino acids, 11 biogenic amines, 24 acylcarnitines, 39 glycerophospholipids and 6 sphingolipids) were significantly different (Kruskal–Wallis p-value < 0.05) across the four groups (Supplementary Table 1). However, after application of a Benjamini–Hochberg correction for multiple testing, 9 metabolites were determined to be significantly lower in TBI groups compared to sham groups (q-value < 0.05) and returned to sham levels with RIC treatment (Table 1; Fig. 2). Further, the biogenic amines and acylcarnitine concentrations were lower in the TBI group compared with shams and were restored to sham levels with RIC treatment. Plasma concentrations of six amino acids (Asn, Pro, Glu, Gly, Thr and Tyr), carnosine and two acylcarnitines (C2 and C3) were higher in TBI RIC compared with TBI (Fig. 2). However, 16 out of 24 acylcarnitines and all 39 glycerophospholipids were lower in RIC groups compared with groups that did not receive RIC (Supplementary Fig. 1). Overall, plasma amino acid metabolites were significantly depleted in the TBI group but not in the TBI RIC intervention group.

**Table 1:** The micromolar concentrations of 94 (50.8%) metabolites (14 amino acids, 11 biogenic amines, 24 acylcarnitines, 39 glycerophospholipids and 6 sphingolipids) were significantly different (p-value < 0.05) across the four groups.

Metabolite	Class	p-value	−log10(p)	q-value
C3	Acylcarnitine	2.36E−4	3.6274	0.021461
Carnosine	Biogenic amine	2.12E−4	3.6738	0.021461
Asn	Amino acid	9.34E−4	3.0278	0.034147
Gly	Amino acid	8.17E−4	3.0877	0.034147
Tyr	Amino acid	6.71E−4	3.1735	0.034147
Thr	Amino acid	1.13E−3	2.9471	0.034265
Glu	Amino acid	1.32E−3	2.8773	0.034490
C2	Acylcarnitine	1.53E−3	2.8151	0.034793
Pro	Amino acid	1.72E−3	2.7643	0.034793

However, after application of nonparametric Kruskal–Wallis test with Benjamini–Hochberg corrections, 9 metabolites were determined to be significantly lower in TBI groups (q-value < 0.05): six amino acids (Asn, Pro, Glu, Gly, Thr and Tyr), carnosine, and two acylcarnitines (C2 and C3). The class, original p-value, -log10(p), and q-value for each significant metabolite are listed.

### Untargeted proteomics identified differentially expressed proteins between brain-injured and RIC-treated mice

**Figure 3:**
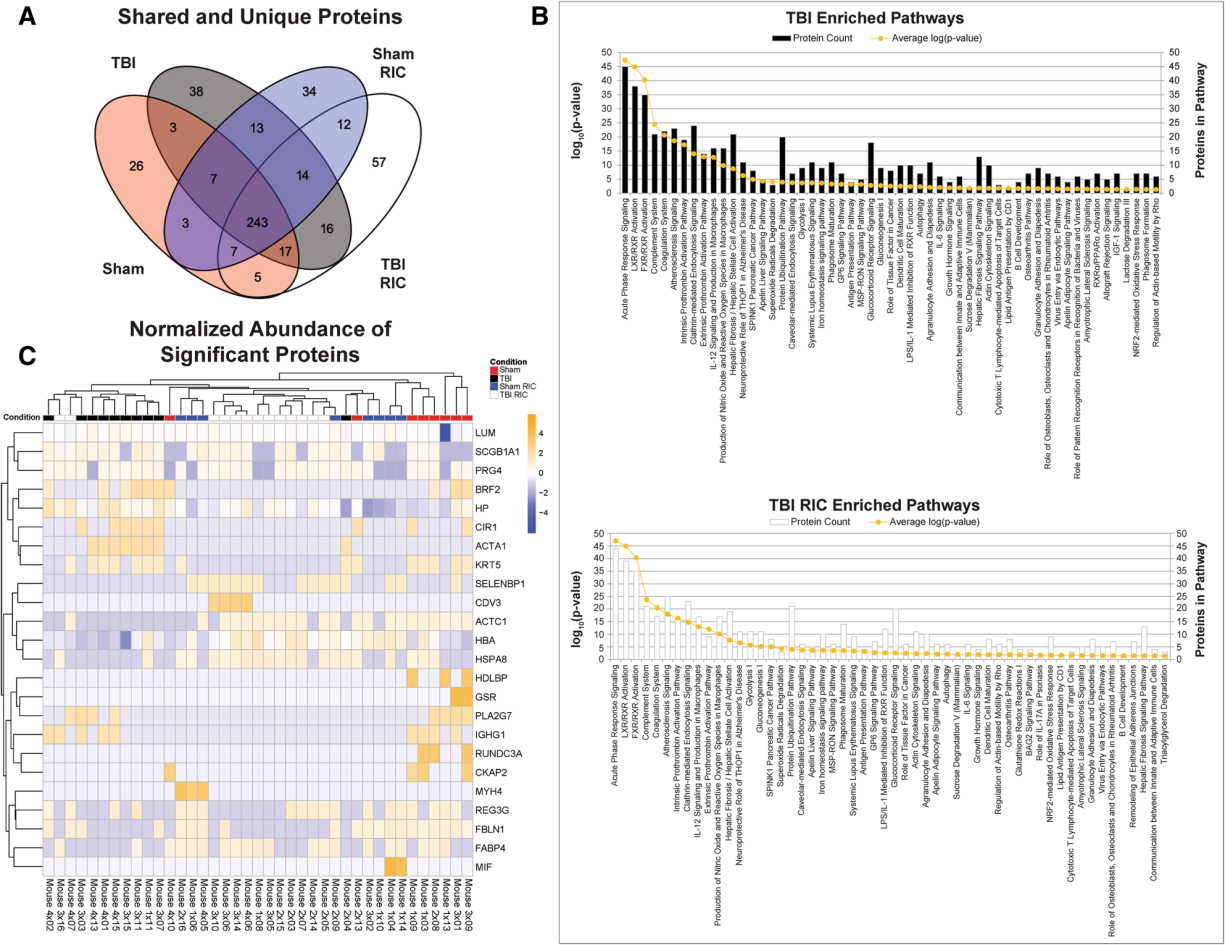
Protein identification in sham, sham RIC, TBI, and TBI RIC conditions from discovery proteomics by liquid chromatography-tandem mass spectrometry. (**A**) Shared and unique proteins detected in each of the 4 brain injury conditions. This analysis identified 493 unique proteins across all four groups. Of these, 243 proteins were shared between brain injury and treatment groups, whereas ~ 7.7% (38) and ~ 11.5% (57) were unique to TBI and TBI RIC groups, respectively. (**B**) Barplot showing the number of proteins mapped to enriched pathways from TBI RIC (black bars) and TBI (white bars) conditions. The orange dots represent the log10(p-value) of the pathway. (**C**) Heatmap of z-scores for significantly differentially abundant proteins (p-value < 0.05) across all mice based on a Kruskal–Wallis test.

Liquid chromatography-tandem mass spectrometry (LC–MS/MS analysis) identified 493 unique proteins across all four groups. Of these, 243 proteins were shared between all 4 groups (Fig. 3A), whereas ~ 8% (38) and ~ 12% (57) were unique to TBI and TBI RIC groups, respectively. Pathway analysis based on the entire proteome revealed 63 uniquely enriched pathways, 46 (73%) of which were shared across all conditions (Fig. 3B, Supplementary Table 2). *BAG2 Signaling pathway* was the only pathway shared between RIC groups*.* Cathepsin B (CTSB), Heat Shock Protein Family A (HSP70) Member 8 (HSPA8), and HSP70 Member 5 (HSPA5) were mapped to BAG2 Signaling pathway in both Sham RIC and TBI RIC. Heat Shock Protein Family A (Hsp70) Member 4 (HSPA4), and proteasome activator subunit 4 (PSME4) mapped from Sham RIC, while HSP70 Member 9 (HSPA9) was only in TBI RIC. *Hepatic Fibrosis Signaling Pathways* was the only pathway found in both TBI groups. Glutathione redox Reactions I, Insulin-like growth factor (IGF)1 signaling, and Lactose degradation III were only enriched in untreated TBI.

To determine candidate biomarkers of TBI and RIC treatment, a non-parametric Kruskal–Wallis test revealed 24 differentially abundant proteins (p-value < 0.05) (Fig. 3C, Table 2). Actin alpha cardiac muscle (ACTC) 1, Hemoglobin Subunit Alpha (HBA), and HSPA8 were 2–fourfold lower in TBI compared to sham and resolved to sham levels with RIC treatment. BRF2 (BRF2 RNA Polymerase III Transcription Initiation Factor Subunit), Haptoglobin (HP), Corepressor Interacting with RBPJ (CIR1), actin, alpha 1, skeletal muscle (ACTA1) were more abundant in the TBI group but lower or undetectable in RIC treated groups. After application of a Benjamini–Hochberg correction for multiple testing, ACTA1 was found to be significantly increased in TBI compared to both sham groups and TBI RIC (q-value < 0.05).

**Table 2:** A Kruskal–Wallis test revealed 24 differentially abundant proteins between the 4 conditions (p-value < 0.05).

Gene	Accession	p-value	q-value
Acta1	P68134	3.72E−06	1.81E−03
Krt5	Q922U2	4.52E−04	1.10E−01
Ckap2	Q3V1H1	7.89E−04	1.28E−01
Actc1	P68033	1.79E−03	1.87E−01
Brf2	Q3UAW9	1.93E−03	1.87E−01
Cir1	Q9DA19	2.78E−03	2.26E−01
Fabp4	P04117	5.09E−03	2.95E−01
Rundc3a	O08576	6.58E−03	2.95E−01
Myh4	Q5SX39	6.58E−03	2.95E−01
Hdlbp	Q8VDJ3	6.58E−03	2.95E−01
Fbln1	Q08879-2	6.91E−03	2.95E−01
Hp	Q61646	7.84E−03	2.95E−01
Hba	P01942	7.88E−03	2.95E−01
Prg4	Q9JM99-3	1.23E−02	4.27E−01
Reg3g	O09049	1.64E−02	5.32E−01
Hspa8	P63017	2.00E−02	5.79E−01
Selenbp1	P17563	2.02E−02	5.79E−01
Pla2g7	Q60963	3.13E−02	7.52E−01
Ighg1	P01869	3.23E−02	7.52E−01
Cdv3	Q4VAA2	3.43E−02	7.52E−01
Lum	P51885	3.62E−02	7.52E−01
Scgb1a1	Q06318	4.29E−02	7.52E−01
Mif	P34884	4.70E−02	7.52E−01
Gsr	P47791	4.70E−02	7.52E−01

After Benjamini–Hochberg correction, ACTA1 was the only protein with q-value < 0.05. Gene names, Uniprot accesions, p-values, and q-values are listed.

## Discussion

The current study established new blood-based monitoring biomarkers that increased in abundance after TBI and returned to basal levels with RIC treatment. Previous studies have shown that RIC intervention can alleviate TBI-induced molecular events in human patients and acute cognitive deficits in a mouse model^24^. Furthermore, RIC intervention can reduce TBI associated pathologies in cases without immediate access to health care at the time of injury, as the procedure only requires a blood pressure cuff and minimal training to perform. To identify molecular mechanisms of pathological processing and track the course of disease in TBI and RIC treatment, metabolomics and proteomic analyses were used in this study. These analyses have been previously shown to be a diagnostic tool in severe TBI^25^.

In this study, the abundance of nine metabolites were significantly changed (p < 0.05) after TBI and RIC treatment: six amino acids (Asn, Pro, Glu, Gly, Thr and Tyr), carnosine, and two acylcarnitines (C2 and C3). In a previous study using a weight drop model of TBI in rats, Asn, Glu, Gly, Thr, Tyr levels were significantly modified in the injured brain compared to sham mice over 5 days post-injury^26^. These amino acids were also significantly higher in plasma at 24-h post-injury compared to shams. Furthermore, Tyr plasma levels were significantly lowered in patients after a severe TBI^27^. Plasma concentrations of Glu did not change at 72 h after lateral FPI in the rat^28^. However, Pro levels were significantly lower at 24 h after lateral FPI and remained low up to 72 h; reductions in Pro negatively correlated with functional outcome measured by a neurological severity score^28^. In the current study, RIC treatment after TBI restored Pro levels in plasma to sham levels.

Previously, in a rat model of remote ischemic preconditioning (RIPC), plasma Gly and carnosine levels differentiated RIPC-treated and non-treated groups^29^. This increase in carnosine and Gly may play a role in the mechanism of RIC treatment’s neuroprotective effect. Low levels of Gly in the brain were associated with an increase in neuronal injury after ischemic injury, while increased Gly in the same model was neuroprotective^30^. In another study, intraperitoneal administration of carnosine reduced brain edema and blood brain barrier permeability in a rat model of subarachnoid hemorrhage^31,32^. Other metabolites associated with RIC treatment not identified in this study as differentially abundant or were not measured include ornithine (decreased with RIPC), kynurenine, spermine, and serotonin (increased with RIPC)^29^. Ornithine, putrescine, spermidine and spermine are synthesized from arginine. In our study, we observed increased putrescine and spermidine following RIC in agreement with the RIPC study. Cytotoxic edema is the most immediate symptom after TBI which results in the release of osmolytes, such as taurine^33^. We observed higher concentrations of taurine after TBI compared with shams and these concentrations were lowered in TBI mice that received RIC intervention compared with TBI mice that did not receive RIC. Although, we observed higher concentrations of taurine in the plasma of TBI mice, others have shown elevated levels of taurine in cerebrospinal fluid^33^. As these biofluids do have different properties and biological functions, metabolites may be degraded or differentially metabolized in the cerebral spinal fluid (CSF) compared to blood.

This study identified 24 potential protein biomarkers, one which was significantly increased only in TBI after Benjamini–Hochberg correction. Previous proteomic studies in TBI and stroke in CSF and brain tissue found changes in levels in actin, alpha skeletal muscle. In a previous study in a mouse model of stroke, ACTA1 was twofold lower in the brain compared to sham controls within 2 h after injury^34^. In a study using CSF from TBI patients, actin, alpha skeletal muscle (ACTS) was one of 130 proteins identified using shotgun proteomics, a proteomics technique that covers a wider range of potential protein markers^35^. Future studies have substantial complementary evidence to validate ACTA1 as a biomarker of TBI.

This study identified enriched pathways from proteomics analysis and revealed *BAG2 Signaling pathway* as commonly enriched between RIC groups, involving CTSB, HSPA4, HSPA8, HSPA5, and PSME4 proteins. HSPA members have been reported to facilitate the proper folding of newly translated proteins and stabilize or degrade misfolded or mutant proteins^36^. Members of the heat shock protein 70 family and have also been linked to cancer and multiple neurodegenerative diseases^37^. Overexpression of HSP70 suppressed ischemia–reperfusion damage, as well as Alzheimer’s disease, Parkinson’s disease, and Huntington’s disease^38–40^. One study found that HSPA8 functions as a safeguard for protein homeostasis and is reduced in brains of Alzheimer’s disease, Parkinson’s disease, and Huntington’s disease^41^. In this study, RIC treatment after diffuse TBI increased HSPA8 in the discovery phase. RIC may act on the *BAG2 Signaling pathway* and is a potential mechanistic pathway for TBI-induced neurodegenerative disease processes**.**

G*lutathione redox reactions I* was enriched in every group except TBI. Glutathione is a thiol that is an anti-oxidant against reactive oxygen and nitrogen species. Glutathione precursors can also protect the brain from trauma^42^. In clinical studies, glutathione levels were lower in CSF of pediatric TBI patients compared with healthy controls between 1 and 7 days post-injury^42,43^. Experimental TBI in rats was associated with a reduction of glutathione in the brain, where the lowest concentration occurred within 24–48-h post-TBI, and was associated with increased susceptibility of neuronal damage by free radicals^42,44^. Changes in proteins associated with the glutathione pathway may be a potential therapeutic pathway for TBI. Additionally, the *IGF-1 signaling* pathway was identified as a potential therapeutic target, as it was only enriched in TBI. IGF-1 is a hormone that regulates brain plasticity^45^. Exogenous increases of IGF-1 promotes neuronal survival after trauma and may be neuroprotective^45^. In TBI patients, serum levels of IGF-1 were lower than in controls^45–49^. In mouse models of TBI (cortical contusion), IGF-1 was increased in brain tissue^50^. Since *IGF-1 signaling* was only enriched in the TBI group and not in either the Sham groups or TBI RIC groups, the RIC intervention may prevent TBI-induced changes in IGF-1. These data suggest that the most promising TBI monitoring biomarkers may be associated with the IGF-1 pathway and proteins necessary for glutathione redox reactions.

One objective in the current study was to discover potential monitoring biomarkers for TBI and RIC efficacy. Commercial antibodies for Acta1 Elisa/western blot are currently unavailable, thus we were unable to validate the untargeted proteomic results. Another limitation to this study is the exclusion of females. Females and males respond differently to both TBI and RIC^51–54^. Therefore, one sex (male) was used to reduce variability and sex differences. Further studies will determine whether the biomarkers found for RIC and TBI in this study can extend to females. Many TBI-induced molecular mechanisms are likely engaged immediately after injury. Our study design was limited to one-day post-TBI and focused on these short-term effects. Future studies will focus on mid and long-term effects to study delayed mechanisms related to RIC and TBI. This study has elucidated mechanisms for TBI and RIC treatment in an unbiased manner. Six amino acids (Asn, Pro, Glu, Gly, Thr and Tyr), carnosine, and two acylcarnitines (C2 and C3) may act as potential monitoring biomarkers as well as ACTA1 protein levels in plasma. Furthermore, pathway analysis revealed unique pathways to RIC treated groups and TBI groups that may point to mechanisms of therapeutic efficacy for RIC intervention. Using proteins and metabolites as potential biomarkers for brain injury should be more reliable to determine injury and recovery than using behavioral outcomes as biomarkers. Biological monitoring biomarkers is necessary to improve clinical care of a positive TBI diagnosis. Further studies will be necessary to determine which mechanisms mediate therapeutic efficacy after TBI. The identified metabolites and proteins provide molecular mechanisms for TBI and therapeutic RIC in order to monitor disease progression and therapeutic efficacy.

## Materials and methods

### Animals

Male C57BL/6 mice (20–24 g; Harlan Laboratories, Inc., Indianapolis, IN) were group housed for all studies. Mice were housed in a 14 h light/10 h dark cycle at a constant temperature (23 ± 2 °C) with food and water available ad libitum according to the Association for Assessment and Accreditation of Laboratory Animal Care International. Mice were acclimated to their environment following shipment for at least three days prior to any experiments. Animal care was approved by the University of Arizona Institutional Animal Care and Use Committee. Study design is shown in Fig. 1A. There were 39 mice in the discovery group [sham (n = 8), TBI (n = 10), sham RIC (n = 8), TBI RIC (n = 13)]. Cheek bleeds were performed 24 h after RIC procedure, collected into EDTA-coated microcentrifuge tubes and immediately stored on ice. Plasma was separated by centrifugation at 3,000×*g* for 10 min and immediately stored at -80 °C for downstream metabolomic and proteomic analyses.

### Diffuse traumatic brain injury by midline fluid percussion injury

Standard procedures were used to administer midline fluid percussion injury (mFPI) or sham injury to young adult (8–10 weeks of age) male mice (20–24 g)^21^. Briefly, mice were anesthetized using 5% isoflurane in 100% oxygen for five minutes, then placed in a stereotaxic frame with continuously delivered isoflurane at 2.5%. While anesthetized, body temperature was maintained using a Deltaphase isothermal heating pad (Braintree Scientific Inc., Braintree, MA). Following a midline incision, a 3 mm craniectomy was performed midway between bregma and lambda using a trephine. A modified Leur-Loc needle hub (3 mm inside diameter) was placed over the exposed intact dura and surrounded by dental acrylic. The incision was sutured at the anterior and posterior edges and topical Lidocaine ointment was applied. The injury hub was closed using a Luer-Loc cap and mice were placed in a heated recovery cage. Mice were monitored until ambulatory before being returned to their home cage overnight before injury induction.

Approximately 24-h post-surgery, mice were re-anesthetized with 5% isoflurane delivered for five minutes. The cap was removed from the injury-hub assembly and the dura was visually inspected through the hub for damage. The hub was then filled with saline and attached to an extension tube connected to the fluid percussion device (Custom Design and Fabrication, Virginia Commonwealth University, Richmond, VA). The pendulum was dropped from a pre-determined angle to induce a diffuse TBI (approximately 1.2–1.4 atmospheres of pressure). Animals in the sham group were re-anesthetized and connected to the injury device; however, no fluid pulse was delivered. Shams righted within 20 s to be included in the study, and all shams were included. Mice were monitored for the presence of a forearm fencing response and righting reflex times were recorded for the injured mice as indicators of injury severity^55^. The righting reflex time is defined as the total time from the initial impact until the mouse spontaneously rights itself from a supine position. The fencing response is defined as a tonic posturing characterized by extension and flexion of opposite arms that has been validated as an overt indicator of injury severity^55^. The injury hub was removed, and the brain was inspected for uniform herniation, hematoma, and integrity of the dura. The dura was intact in all mice; none were excluded as technical failures. The incision was cleaned using saline and closed using sutures. Brain-injured mice had righting reflex recovery times greater than six minutes and a positive fencing response. After spontaneously righting, mice were placed in a heated recovery cage and monitored until ambulatory before being returned to their home cage. After injuries, mice were evaluated by a physical examination and documentation of each animal’s condition including weight.

### Remote ischemic conditioning (RIC)

RIC was administered 1-h following recovery of righting reflex after mFPI. Mice were anesthetized using 5% isoflurane in 100% oxygen for three minutes and placed in a supine position. Light anesthesia was maintained throughout the RIC procedure using 1.5% isoflurane in 100% oxygen via nosecone. RIC was applied using a disposable plastic-coated wire tie (100 mm length × 2 mm diameter). With the left mouse hind limb extended at the knee, a wire tie was wrapped around the hind limb as proximally to the hip as possible. Pressure was applied by twisting the wire tie approximately four times clockwise. RIC was administered in four cycles composed of 5 min RIC and 5 min reperfusion (procedure duration of 40 min). Mice were monitored while recovering from anesthesia before being returned to their home cage. Sham and TBI mice not assigned to a RIC group received equal anesthesia treatment without wire tie application.

### Metabolomics analysis

Targeted metabolomics were performed on 185 metabolites (21 amino acids, 21 biogenic amines, 88 glycerophospholipids, 14 sphingolipids, 40 acylcarnitines, 1 hexose) using the AbsoluteIDQ p180 kit (Biocrates Life Sciences AG, Innsbruck, Australia). The metabolites were extracted from 10 µL of plasma as per manufacturer’s protocol. Seven samples were not included in only the metabolomics section of the study due to high hemolytic scores or low volume (2 TBI, 5 TBI RIC). Data acquisition was carried out on an Acquity UPLC and Xevo-TQ-S mass spectrometer (Waters, Milford, MA) using reversed phase separation and flow injection analysis as per vendor’s instructions. The standard curve and quality controls were within the acceptance criteria recommended by the Federal Drug Administration (FDA; Guidance of Industry Bioanalytical Method Validation). A pooled sample was employed for quality control (QC), and only metabolites measured in all QC samples with less than 20% coefficient of variation (CV) were included in the analysis. All samples were randomized during preparation and data acquisition. All concentrations were measured in micromolar. For this analysis, lipids included lysophosphatidyl glycerophospholipids (LysoPC x:y), glycerophospholipids (PC aa x:y and PC ae x:y) and sphingolipids (SM x:y, SM[OH] x:y), where x denotes the number of carbons in the side chain and y represents number of unsaturated chains.

### Proteomics analysis

High abundance proteins were removed from plasma using Seppro Mouse M7 spin columns (Sigma-Aldrich, St. Louis, Mo). Protein concentration was quantified using Pierce BCA (Thermo Fisher Scientific, Waltham, MA). Equal amounts of protein were reduced using 10 mM dithiothreitol (1 h), alkylated using 40 mM iodoacetamide and digested overnight at 37ºC using Trypsin Gold at a 1:20 protein to enzyme ratio (Promega Corporation, Madison, WI). Samples were subjected to solid phase extraction using 1 cc SEP-PAK C18 (Waters), vacuum-dried and frozen at − 80 °C until LC–MS/MS analysis.

Peptides were reconstituted in 0.1% formic acid for analysis as previously described. LC–MS/MS data were acquired on an Orbitrap Fusion Lumos tribrid mass spectrometer (Thermo, San Jose, CA) coupled to a nanoAcquity UPLC system (Waters, Millford, MA). One µg of peptides was loaded on a trapping column (Thermo Scientific Acclaim PepMap 100 C18, 75 µm, 2 cm, 3 µm particle size, 100 Å pore size) and washed for 10 min with 99.5% solvent A (0.1% formic acid in water) and 0.5% solvent B (0.1% formic acid in acetonitrile) at a flow rate of 4 µL/min. The trapped peptides were separated using an Acclaim PepMap RSLC C18 analytical column (Thermo Scientific, 50 µm, 15 cm, 2 µm particle size, 100 Å pore size) and a 95 min gradient (3% to 7% solvent B for 1 min, 7% to 25% solvent B for 72 min, 25% solvent B to 45% B for 10 min, 45% to 90% B for 0.5 min, 90% B for 1 min, 3% B in 0.5 min and re-equilibration at 3% B for 10 min) at 300 nL/minute flow rate. Data-dependent acquisition was performed in Top Speed mode with a duty cycle of 3 s and the following parameters: spray voltage of 2100 V, ion transfer tube temperature of 275 °C, survey scan in the Orbitrap at a resolution of 120 K at 200 m/z, scan range of 400–1,500 m/z, AGC target of 2E5 and maximum ion injection time of 50 ms. Parent scans were followed by a daughter scan using High Energy Collision (HCD) dissociation of top abundant peaks and detection in the ion-trap with the following settings: quadrupole isolation mode, isolation window at 1.4 m/z, AGC target of 5E3 with maximum ion injection time of 35 ms and 35% HCD collision energy^56^. Dynamic exclusion was set to 60 s. Peptide precursor ion intensities were used to calculate relative abundance. Protein identification and relative quantification was conducted with Proteome Discoverer v2.1 (Thermo Fisher Scientific, Waltham, MA) and Mascot v2.3 (Matrix Science, Boston, MA) using the Mouse UniprotKB/SwissProt 2015 database. Protein abundances were normalized using the DEP package in R. Pathway analysis was performed using Ingenuity Pathway Analysis (IPA, Qiagen) and the Ingenuity Knowledgebase (mouse) with default settings to elucidate enriched biological pathways in each condition.

### Statistics

Righting reflex times were analyzed using a two-tailed unpaired t-test. For mass spectrometry analyses, Gaussian distribution was verified using density, q-q plots and Shapiro–Wilk’s tests. For metabolomics and proteomics analyses, a nonparametric Kruskal–Wallis test with Benjamini–Hochberg corrections for multiple testing was used to determine significance. A post-hoc test was used to calculate pairwise differences between groups, for molecules with q-value < 0.05. For multiple group comparisons in metabolomics, metabolites with more than 50% missing values in each of the experimental groups were excluded from analysis.

### Ethics approval and informed consent

All animal studies were conducted in accordance with the guidelines established by the internal IACUC (Institutional Animal Care and Use Committee) and the NIH guidelines for the care and use of laboratory animals.

## Supplementary information

Supplementary file 1.
