# Supplementary material for: Proteomic analysis identifies plasma correlates of remote ischemic conditioning in the context of experimental traumatic brain injury
Source: Sci Rep. 2020 Jul 31;10:12989. doi: 10.1038/s41598-020-69865-4 (PMC7395133; doi:10.1038/s41598-020-69865-4)
Supplement: Supplementary file 1 [file 41598_2020_69865_MOESM1_ESM.pdf]

Title:

Proteomic analysis identifies plasma correlates of remote ischemic conditioning in the context of experimental traumatic brain injury

Authors: Maha Saber[1,2], Khyati V Pathak[3], Marissa McGilvrey[3], Krystine Garcia-Mansfield[3], Jordan L Harrison[1,2], Rachel K Rowe[1,2,4], Jonathan Lifshitz[1,2,4+], Patrick Pirrotte[3+]

Keywords: Remote Ischemic Conditioning, Traumatic Brain Injury, Proteomics, Metabolomics, Biomarkers

Affiliations:

[1]BARROW Neurological Institute at Phoenix Children's Hospital, Phoenix, AZ

[2]Child Health, University of Arizona College of Medicine – Phoenix, Phoenix, AZ

[3]Collaborative Center for Translational Mass Spectrometry, Translational Genomics Research Institute, Phoenix, AZ

[4]Phoenix VA Health Care System, Phoenix, AZ

[+]These authors share senior authorship of this manuscript

Corresponding Author:

Jonathan Lifshitz, Ph.D.

University of Arizona College of Medicine-Phoenix

Barrow Neurological Institute at Phoenix Children's Hospital

425 N 5th street ABC1

Phoenix, AZ 85004, USA

Phone: 602-827-2346

Email: jlifshitz@email.arizona.edu

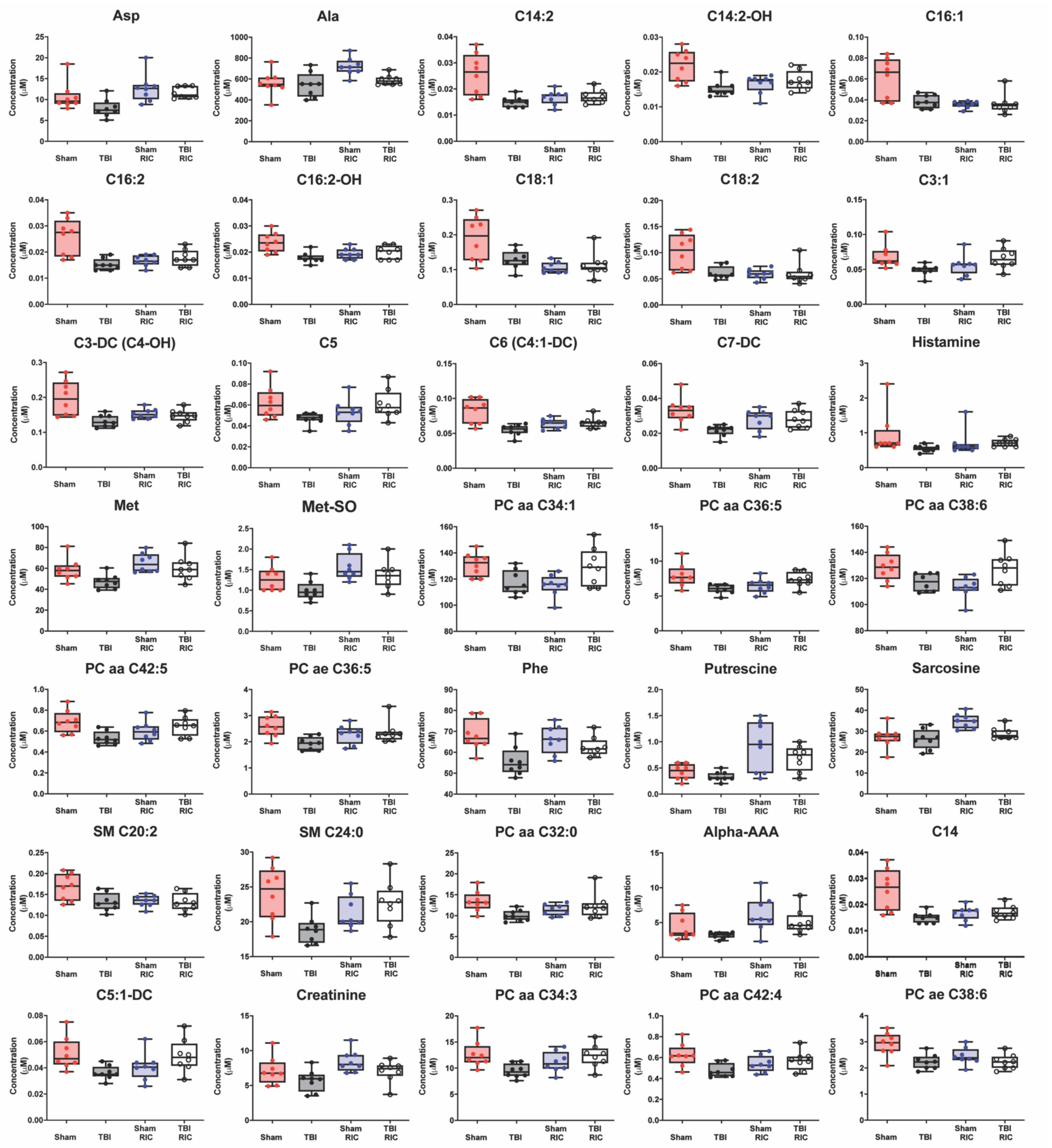
Asp
Ala
C14:2
C14:2-OH
C16:1
C16:2
C16:2-OH
C18:1
C18:2
C3:1
C3-DC (C4-OH)
C5
C6 (C4:1-DC)
C7-DC
Histamine
Met
Met-SO
PC aa C34:1
PC aa C36:5
PC aa C38:6
PC aa C42:5
PC ae C36:5
Phe
Putrescine
Sarcosine
SM C20:2
SM C24:0
PC aa C32:0
Alpha-AAA
C14
C5:1-DC
Creatinine
PC aa C34:3
PC aa C42:4
PC ae C38:6
Concentration (μM)
Sham
TBI
Sham RIC
TBI RIC

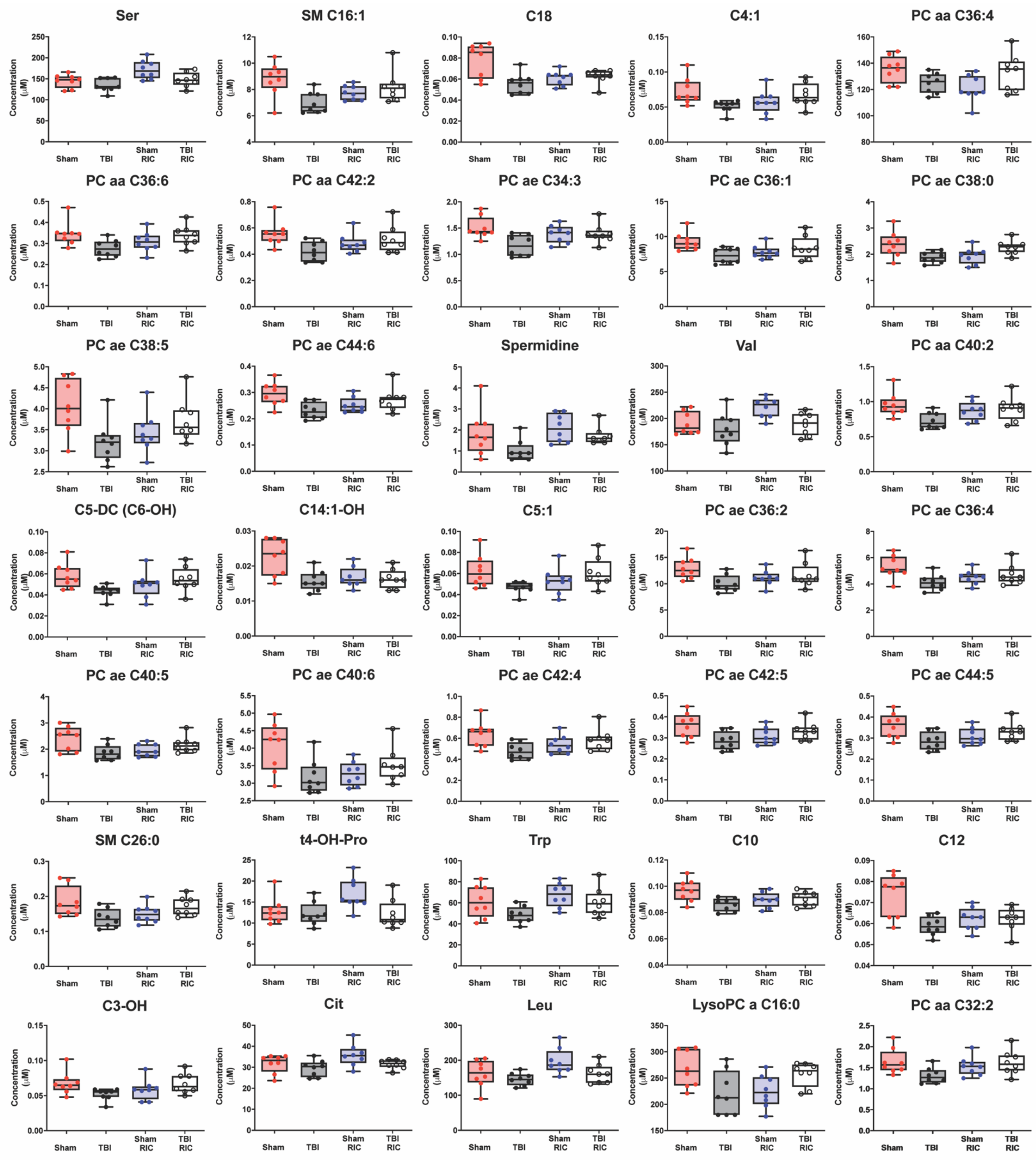
Ser
SM C16:1
C18
C4:1
PC aa C36:4
PC aa C36:6
PC aa C42:2
PC ae C34:3
PC ae C36:1
PC ae C38:0
PC ae C38:5
PC ae C44:6
Spermidine
Val
PC aa C40:2
C5-DC (C6-OH)
C14:1-OH
C5:1
PC ae C36:2
PC ae C36:4
PC ae C40:5
PC ae C40:6
PC ae C42:4
PC ae C42:5
PC ae C44:5
SM C26:0
t4-OH-Pro
Trp
C10
C12
C3-OH
Cit
Leu
LysoPC a C16:0
PC aa C32:2
Concentration (μM)
Sham
TBI
Sham RIC
TBI RIC

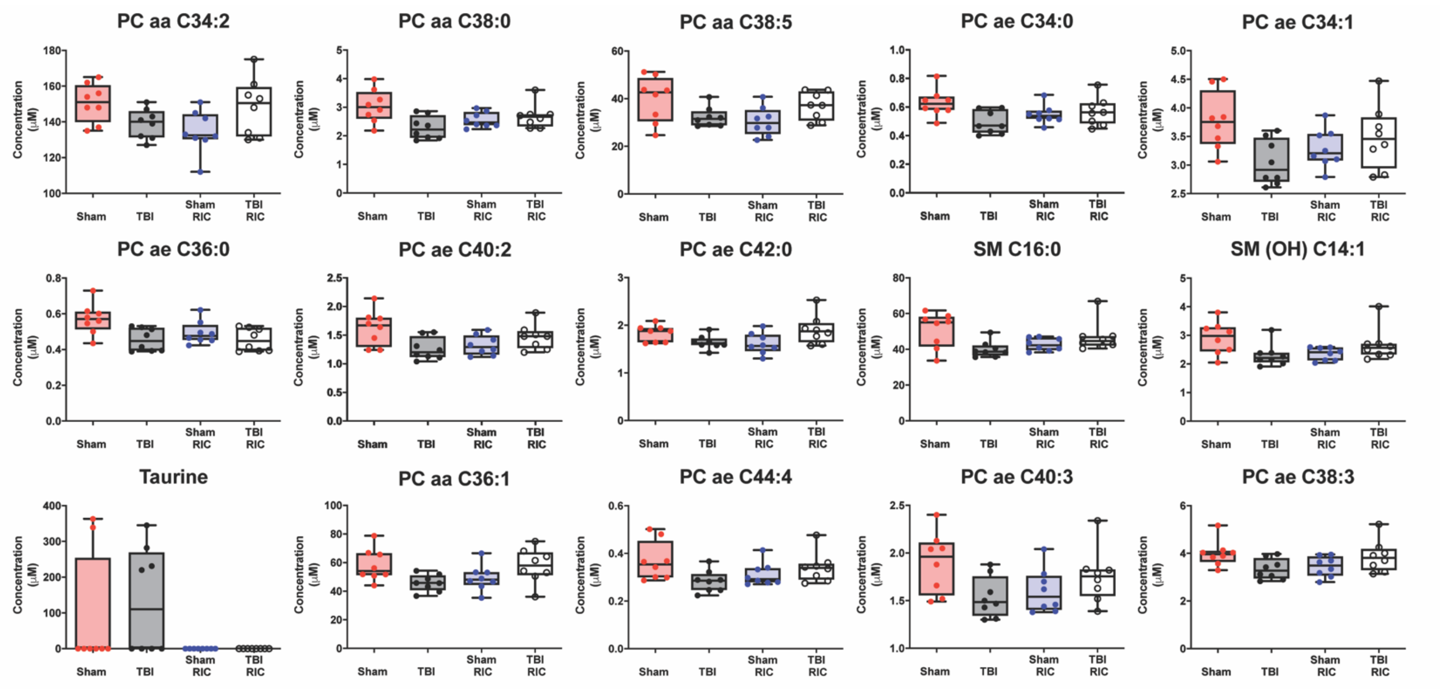

Supplementary Figure 1: Box-Whisker plots representing concentrations of significantly different metabolites between sham, TBI, sham RIC and TBI RIC mice. The targeted metabolomics analysis was performed on plasma obtained from mice subjected to sham, TBI, sham RIC and TBI-RIC treatments. The concentrations of metabolites were measured in μM unit. The micromolar concentrations of 94 (50.8%) metabolites (14 amino acids, 11 biogenic amines, 24 acylcarnitines, 39 glycerophospholipids and 6 sphingolipids) were significantly different (p-value < 0.05) across the four groups, prior to Kruskal-Wallis test with Benjamini-Hochberg corrections.

Metabolite	Class	p-value	-LOG10(p)	q-value
Asn	Amino acid	0.0009381	3.0278	0.034147
Pro	Amino acid	0.0017205	2.7643	0.034793
Tyr	Amino acid	0.00067065	3.1735	0.034147
Thr	Amino acid	0.0011296	2.9471	0.034265
Glu	Amino acid	0.0013265	2.8773	0.03449
Gly	Amino acid	0.00081723	3.0877	0.034147
Carnosine	Biogenic amine	0.00021193	3.6738	0.021461
C2	Acylcarnitine	0.0015309	2.8151	0.034793
C3	Acylcarnitine	0.00023584	3.6274	0.021461
Asp	Amino acid	0.0032577	2.4871	0.05929
Ala	Amino acid	0.010588	1.9752	0.064175
C14:2	Acylcarnitine	0.0063688	2.1959	0.064175
C14:2-OH	Acylcarnitine	0.011308	1.9466	0.064175
C16:1	Acylcarnitine	0.0081492	2.08895	0.064175
C16:2	Acylcarnitine	0.0046298	2.3344	0.064175
C16:2-OH	Acylcarnitine	0.0089009	2.0506	0.064175
C18:1	Acylcarnitine	0.0078919	2.1028	0.064175
C18:2	Acylcarnitine	0.0069747	2.1565	0.064175
C3:1	Acylcarnitine	0.012119	1.9165	0.064175
C3-DC(C4-OH)	Acylcarnitine	0.011398	1.9432	0.064175
C5	Acylcarnitine	0.0047227	2.3258	0.064175
C6 (C4:1-DC)	Acylcarnitine	0.0075964	2.1194	0.064175
C7-DC	Acylcarnitine	0.010841	1.9649	0.064175
Histamine	Biogenic amine	0.012341	1.9086	0.064175
Met	Amino acid	0.010285	1.9878	0.064175
Met-SO	Biogenic amine	0.0074966	2.1251	0.064175
PC aa C341	Glycerophospholipid	0.01179	1.9285	0.064175
PC aa C365	Glycerophospholipid	0.01218	1.9144	0.064175

PC aa C386	Glycerophospholipid	0.010327	1.986	0.064175
PC aa C425	Glycerophospholipid	0.0086017	2.0654	0.064175
PC ae C365	Glycerophospholipid	0.00901	2.0453	0.064175
Phe	Amino acid	0.011216	1.9501	0.064175
Putrescine	Biogenic amine	0.0089338	2.049	0.064175
Sarcosine	Biogenic amine	0.0038914	2.4099	0.064175
SM C202	Sphingolipid	0.005466	2.2623	0.064175
SM C240	Sphingolipid	0.01357	1.8674	0.068605
PC aa C320	Glycerophospholipid	0.01412	1.8502	0.069457
alpha-AAA	Biogenic amine	0.015727	1.8034	0.072329
C14	Acylcarnitine	0.017985	1.7451	0.072329
C51-DC	Acylcarnitine	0.016535	1.7816	0.072329
Creatinine	Biogenic amine	0.016335	1.7869	0.072329
PC aa C343	Glycerophospholipid	0.017832	1.7488	0.072329
PC aa C424	Glycerophospholipid	0.017178	1.765	0.072329
PC ae C386	Glycerophospholipid	0.018281	1.738	0.072329
Ser	Amino acid	0.017098	1.7671	0.072329
SM C161	Sphingolipid	0.016513	1.7822	0.072329
C18	Acylcarnitine	0.024637	1.6084	0.077397
C41	Acylcarnitine	0.022191	1.6538	0.077397
PC aa C364	Glycerophospholipid	0.024227	1.6157	0.077397
PC aa C366	Glycerophospholipid	0.02415	1.6171	0.077397
PC aa C422	Glycerophospholipid	0.023678	1.6257	0.077397
PC ae C343	Glycerophospholipid	0.020682	1.6844	0.077397
PC ae C361	Glycerophospholipid	0.024162	1.6169	0.077397
PC ae C380	Glycerophospholipid	0.023322	1.6322	0.077397
PC ae C385	Glycerophospholipid	0.023005	1.6382	0.077397
PC ae C446	Glycerophospholipid	0.024665	1.6079	0.077397
Spermidine	Biogenic amine	0.021189	1.6739	0.077397

Val	Amino acid	0.021982	1.6579	0.077397
PC aa C402	Glycerophospholipid	0.027689	1.5577	0.085414
C5-DC C6-OH	Acylcarnitine	0.028187	1.55	0.085499
C141-OH	Acylcarnitine	0.030046	1.5222	0.089327
C51	Acylcarnitine	0.03268	1.4857	0.089327
PC ae C362	Glycerophospholipid	0.034056	1.4678	0.089327
PC ae C364	Glycerophospholipid	0.034948	1.4566	0.089327
PC ae C405	Glycerophospholipid	0.033188	1.479	0.089327
PC ae C406	Glycerophospholipid	0.03169	1.4991	0.089327
PC ae C424	Glycerophospholipid	0.034369	1.4638	0.089327
PC ae C425	Glycerophospholipid	0.0334	1.4763	0.089327
PC ae C445	Glycerophospholipid	0.032205	1.4921	0.089327
SM C260	Sphingolipid	0.035338	1.4518	0.089327
t4-OH-Pro	Biogenic amine	0.034209	1.4659	0.089327
Trp	Amino acid	0.033965	1.469	0.089327
C10	Acylcarnitine	0.043158	1.3649	0.089362
C12	Acylcarnitine	0.037655	1.4242	0.089362
C3-OH	Acylcarnitine	0.043624	1.3603	0.089362
Cit	Amino acid	0.04337	1.3628	0.089362
Leu	Amino acid	0.042462	1.372	0.089362
lysoPC a C160	Glycerophospholipid	0.038839	1.4107	0.089362
PC aa C322	Glycerophospholipid	0.043909	1.3574	0.089362
PC aa C342	Glycerophospholipid	0.040876	1.3885	0.089362
PC aa C380	Glycerophospholipid	0.038487	1.4147	0.089362
PC aa C385	Glycerophospholipid	0.041223	1.3849	0.089362
PC ae C340	Glycerophospholipid	0.041873	1.3781	0.089362
PC ae C341	Glycerophospholipid	0.03622	1.441	0.089362
PC ae C360	Glycerophospholipid	0.04088	1.3885	0.089362
PC ae C402	Glycerophospholipid	0.036552	1.4371	0.089362

PC ae C420	Glycerophospholipid	0.042827	1.3683	0.089362
SM C160	Sphingolipid	0.040629	1.3912	0.089362
SM OH C141	Sphingolipid	0.041524	1.3817	0.089362
Taurine	Biogenic amine	0.04419	1.3547	0.089362
PC aa C361	Glycerophospholipid	0.045066	1.3462	0.089378
PC ae C444	Glycerophospholipid	0.04518	1.3451	0.089378
PC ae C403	Glycerophospholipid	0.047545	1.3229	0.093046
PC ae C383	Glycerophospholipid	0.048327	1.3158	0.093568

Supplementary Table 1: All 94 metabolites identified via targeted quantitative metabolomics using mass spectrometry are listed including name, class, unadjusted p-value, -LOG10(p), and q-value.

		Sham			TBI			Sham RIC			TBI RIC		
**Canonical Pathway**	**Average log(p-value)**	**Molecules**	**Occurrences**	**Average log(p-value)**	**Molecules**	**Occurrences**	**Average log(p-value)**	**Molecules**	**Occurrences**	**Average log(p-value)**	**Molecules**	**Occurrences**	**Metabolites**
Actin Cytoskeleton Signaling	1.98625	ACTB, I3ACTC, F2, FN1, GSN, KNG1, LBP, MYH1, PFN1	8	1.7889	ACTA1, ACTB, F2, FN1, GSN, KNG1, CFL1, LBP, PFN1, MYH1	10	2.05375	ACTB, ACTC1, F2, FN1, GSN, KNG1, LBP, MYH4, CFL1	8	2.317692308	ACTB, ACTC1, F2, FN1, GSN, KNG1, CFL1, LBP, MYH1, VCL, PFN1	13	
Acute Phase Response Signaling	47.125	AGT, AHSG, ALB, AMBP, APCS, APOA1, APOA2, APOH, C1R, C2, C3, C4A/C4B, C5, C9, CFB, CP, CRP, F2, FGA, FGB, FGG, FN1, HP, HPX, IL1RAP, ITIH2, ITIH3, ITIH4, KLKB1, PLG, RBP4, SAA1, SAA2-SAA4, SERPINA1, SERPINA3, SERPIND1, SERPINF1, SERPINF2, SERPING1, TF, TTR, LBP, MBL2, A2M	8	47.27	A2M, AGT, AHSG, ALB, AMBP, APCS, APOA1, APOA2, APOH, C1R, C2, C3, C4A/C4B, C5, C9, CFB, CP, CRP, F2, FGA, FGB, FGG, FN1, HP, HPX, IL1RAP, ITIH2, ITIH4, KLKB1, PLG,RBP4, SAA1, SAA2-SAA4, SERPINA1, SERPINA3, SERPINF1, SERPINF2, SERPING1, TF, TTR, ITIH3, LBP, MBL2, SERPIND1, C1S	10	47.95	AGT, AHSG, ALB, AMBP, APCS, APOA1, APOA2, APOH, C1R, C1S, C2, C3, C4A/C4B, C5, C9, CFB, CP, CRP, F2, FGA, FGB, FGG, FN1, HP, HPX, IL1RAP, ITIH2, ITIH3, ITIH4, KLKB1, MBL2, PLG, RBP4, SAA1, SAA2-SAA4, SERPINA1, SERPINA3, SERPIND1, SERPINF1, SERPINF2, SERPING1, TF, TTR, A2M, LBP	8	46.97692308	AGT, AHSG, ALB, AMBP, APCS, APOA1, APOA2, APOH, C1R, C2, C3, C4A/C4B, C5, C9, CFB, CP, CRP, F2, FGA, FGB, FGG, FN1, HP, HPX, IL1RAP, ITIH2, ITIH3, ITIH4, KLKB1, MBL2, PLG, RBP4, SAA1, SAA2-SAA4, SERPINA1, SERPINA3, SERPIND1, SERPINF1, SERPINF2, SERPING1, TF, TTR, A2M, LBP	13	
Agranulocyte Adhesion and Diapedesis	2.27625	ACTB, ACTC1, C5, FN1, ICAM1, SELL, VCAM1, MYH1, PF4	8	2.0446	ACTA1, ACTB, C5, FN1, ICAM1, SELL, VCAM1, PF4, MMP2, MYH1, Ccl8	10	1.77875	ACTB, ACTC1, C5, FN1, MYH4, PF4, SELL, ICAM1, PECAM1	8	2.305384615	ACTB, ACTC1, C5, FN1, ICAM1, MYH1, PF4, SELL, SELP, VCAM1	13	
Allograft Rejection Signaling	1.752	B2M, HLA-A, IGHG1, Ighg2a, Ighg2b	8	1.416	B2M, HLA-A, Ighg2b, IGHG1, Ighg2a	10							
Amyotrophic Lateral Sclerosis Signaling	1.65825	CAT, GPX1, IGF1, SOD1, CYCS, CACNA1A	8	1.4878	CAT, IGF1, SOD1, GPX1, CYCS	10	1.77375	CAT, GPX1, SOD1, IGF1, CYCS	8	1.579230769	CAT, GPX1, SOD1, IGF1, CYCS	13	L-glutamic acid,L-glutamine
Antigen Presentation Pathway	2.585	B2M, HLA-A, PSMB5, PSMB6	8	3.16	B2M, HLA-A, PSMB6, PSMB5	10	2.78	B2M, HLA-A, PSMB5, PSMB6	8	3.094615385	B2M, HLA-A, PSMB5, PSMB6	13	
Apelin Adipocyte Signaling Pathway	1.74475	CAT, GPX1, GPX3, SOD1	8	1.5204	CAT, GPX3, SOD1, GPX1	10	2.28375	CAT, GPX1, GPX3, GSTP1, PRDX6, SOD1	8	2.118461538	CAT, GPX1, GPX3, PRDX6, SOD1, GSTP1	13	
Apelin Liver Signaling Pathway	3.27875	AGT, COL1A1, COL1A2, COL11A2, COL3A1	8	4.32	AGT, COL11A2, COL1A1, COL1A2, COL3A1	10	3.47	AGT, COL1A1, COL1A2, COL3A1, COL11A2	8	3.599230769	AGT, COL1A1, COL1A2, COL11A2, COL3A1	13	
Atherosclerosis Signaling	17.9375	ALB, APOA1, APOA2, APOA4, APOB, APOC4, APOD, APOE, APOM, CLU, COL1A1, COL1A2, ICAM1, LCAT, LYZ, PCYOX1, PON1, RBP4, SERPINA1, VCAM1, COL11A2, PLA2G7, S100A8, COL3A1	8	18.62	ALB, APOA1, APOA2, APOA4, APOB, APOC4, APOD, APOE, APOM, CLU, COL11A2, COL1A1, COL1A2, COL3A1, ICAM1, LCAT, LYZ, RBP4, SERPINA1, PON1, PCYOX1, PLA2G7, VCAM1	10	16.5875	ALB, APOA1, APOA2, APOA4, APOB, APOD, APOE, APOM, CLU, COL1A1, COL1A2, LCAT, PCYOX1, PON1, RBP4, SERPINA1, APOC4, COL3A1, LYZ, ICAM1, COL11A2, S100A8	8	17.93076923	ALB, APOA1, APOA2, APOA4, APOB, APOC4, APOE, CLU, COL1A1, COL1A2, LCAT, PON1, RBP4, SERPINA1, APOD, APOM, COL11A2, ICAM1, LYZ, S100A8, PCYOX1, COL3A1, SELP, VCAM1, PLA2G7	13	
Autoimmune Thyroid Disease Signaling	1.32225	HLA-A, IGHG1, Ighg2a, Ighg2b	8										
Autophagy	2.83	CTSA, CTSB, CTSZ, LAMP1, LAMP2, CTSE, CTSS	8	2.312	CTSA, CTSB, LAMP2, CTSE, CTSZ, LAMP1, CTSD	10	1.771875	CTSB, ATG3, CTSA, LAMP1, LAMP2, CTSE, CTSZ	8	2.109923077	CTSA, CTSB, CTSE, CTSZ, LAMP1, LAMP2	13	
B Cell Development	2.04625	HLA-A, Ighg2a, Ighg2b, IGHM	8	1.708	HLA-A, Ighg2b, IGHM, Ighg2a	10	1.808625	HLA-A, Ighg2a, IGHM, Ighg2b	8	1.477692308	HLA-A, IGHM	13	
BAG2 Signaling Pathway							1.691375	CTSB, HSPA4, HSPA8, PSME4, HSPA5	8	1.766153846	CTSB, HSPA8, HSPA5, HSPA9	13	
Caveolar-mediated Endocytosis Signaling	3.7325	ACTB, ACTC1, ALB, B2M, EGFR, HLA-A	8	3.707	ACTA1, ACTB, ALB, B2M, EGFR, HLA-A, ITGB4	10	3.68125	ACTB, ACTC1, ALB, B2M, EGFR, HLA-A	8	3.678461538	ACTB, ACTC1, ALB, B2M, EGFR, HLA-A	13	
Clathrin-mediated Endocytosis Signaling	14.275	ACTB, ACTC1, ALB, APOA1, APOA2, APOA4, APOB, APOC4, APOD, APOE, APOM, CLU, F2, HSPA8, IGF1, LYZ, PCYOX1, PON1, RBP4, SERPINA1, TF, S100A8	8	14	ACTA1, ACTB, ALB, APOA1, APOA2, APOA4, APOB, APOC4, APOD, APOE, APOM, CLU, F2, HSPA8, IGF1, LYZ, RBP4, SERPINA1, TF, PON1, RPS27A, PCYOX1, ITGB4, TFRC	10	14.05	ACTB, ACTC1, ALB, APOA1, APOA2, APOA4, APOB, APOD, APOE, APOM, CLU, F2, HSPA8, PCYOX1, PON1, RBP4, SERPINA1, TF, APOC4, IGF1, LYZ, S100A8	8	14.69230769	ACTB, ACTC1, ALB, APOA1, APOA2, APOA4, APOB, APOC4, APOE, CLU, F2, HSPA8, PON1, RBP4, SERPINA1, TF, APOD, APOM, IGF1, LYZ, S100A8, PCYOX1, TFRC	13	
Coagulation System	19.85	F12, F13B, F2, FGA, FGB, FGG, KLKB1, KNG1, PLG, SERPINA1, SERPINC1, SERPIND1, SERPINF2, F13A1, A2M, F11	8	20.61	A2M, F12, F13B, F2, FGA, FGB, FGG, KLKB1, KNG1, PLG, SERPINA1, SERPINC1, SERPINF2, F13A1, SERPIND1, F5, F10, F7, F9, PROC, PROS1, TFPI	10	21.65	F10, F12, F13B, F2, F9, FGA, FGB, FGG, KLKB1, KNG1, PLG, PROC, PROS1, SERPINA1, SERPINC1, SERPIND1, SERPINF2, A2M, F11, F13A1	8	20.46923077	F12, F13A1, F13B, F2, FGA, FGB, FGG, KLKB1, KNG1, PLG, SERPINA1, SERPINC1, SERPIND1, SERPINF2, A2M, F5, F11	13	
Communication between Innate and Adaptive Immune Cells	2.21625	B2M, HLA-A, IGHG1, Ighg2a, Ighg2b, IGHM	8	1.883	B2M, HLA-A, Ighg2b, IGHM, IGHG1, Ighg2a	10	1.65	B2M, HLA-A, Ighg2a, IGHM, Ighg2b	8	1.448461538	B2M, HLA-A, IGHM, IGHG1	13	
Complement System	22.9875	C1QB, C1QC, C1R, C2, C3, C4A/C4B, C5, C8A, C8B, C8G, C9, CFB, CFD, CFH, CFI, SERPING1, C1QA, MASP2, MBL2	8	24.43	C1QB, C1R, C2, C3, C4A/C4B, C5, C8A, C8B, C8G, C9, CFB, CFD, CFH, CFI, SERPING1, C1QA, C1QC, MASP2, MBL2, Scgb2b27 (includes others), C1S	10	23.425	C1R, C1S, C2, C3, C4A/C4B, C5, C8A, C8B, C8G, C9, CFB, CFD, CFH, CFI, MASP2, MBL2, SERPING1, C1QA, C1QC, Scgb2b27 (includes others), C1QB	8	23.72307692	C1R, C2, C3, C4A/C4B, C5, C8A, C8B, C8G, C9, CFB, CFD, CFH, CFI, MBL2, Scgb2b27 (includes others), SERPING1, C1QC, MASP2, C1QA, C1QB, C1QBP	13	
Crosstalk between Dendritic Cells and Natural Killer Cells	1.436	ACTB, ACTC1, HLA-A, IL2RG	8				1.302	ACTB, ACTC1, HLA-A, IL2RG	8				
Cytotoxic T Lymphocyte-mediated Apoptosis of Target Cells	1.73625	B2M, HLA-A, CYCS	8	1.78	B2M, HLA-A, CYCS	10	1.61625	B2M, HLA-A, CYCS	8	1.600769231	B2M, HLA-A, CYCS	13	
Dendritic Cell Maturation	2.29375	B2M, COL1A1, COL1A2, HLA-A, ICAM1, IGHG1, Ighg2a, Ighg2b, COL11A2, COL3A1	8	2.473	B2M, COL11A2, COL1A1, COL1A2, COL3A1, HLA-A, ICAM1, Ighg2b, IGHG1, Ighg2a	10	1.79	B2M, COL1A1,COL1A2, HLA-A, COL3A1, Ighg2a, ICAM1, Ighg2b, COL11A2	8	1.867923077	B2M, COL1A1, COL1A2, HLA-A, COL11A2, ICAM1, COL3A1, IGHG1	13	
Extrinsic Prothrombin Activation Pathway	11.47875	F12, F13B, F2,FGA, FGB, FGG, SERPINC1, F13A1	8	12.95	F12, F13B, F2,FGA, FGB, FGG, SERPINC1, F13A1, F5, F10, F7, PROC, PROS1, TFPI	10	12.5625	F10, F12, F13B, F2, FGA, FGB, FGG, PROC, PROS1, SERPINC1, F13A1	8	11.9	F12, F13A1, F13B, F2, FGA, FGB, FGG, SERPINC1, F5	13	
FXR/RXR Activation	40.75	AGT, AHSG, ALB, AMBP, APOA1, APOA2, APOA4, APOB, APOC4, APOD, APOE, APOH, APOM, C3, C4A/C4B, C9, CLU, FETUB, FGA, GC, HPX, ITIH4, KNG1, LCAT, PCYOX1, PLTP, PON1, RBP4, SAA1, SERPINA1, SERPINF1, SERPINF2, TF, TTR, VTN, FBP1	8	40.28	AGT, AHSG, ALB, AMBP, APOA1, APOA2, APOA4, APOB, APOC4, APOD, APOE, APOH, APOM, C3, C4A/C4B, C9, CLU, FETUB, FGA, GC, HPX, ITIH4, KNG1, LCAT, PLTP, RBP4, SAA1, SERPINA1, SERPINF1, SERPINF2, TF, TTR, VTN, PON1, PCYOX1	10	41.75	AGT, AHSG, ALB, AMBP, APOA1, APOA2, APOA4, APOB, APOD, APOE, APOH, APOM, C3, C4A/C4B, C9, CLU, FBP1, FETUB, FGA, GC, HPX, ITIH4, KNG1, LCAT, PCYOX1, PLTP, PON1, RBP4, SAA1, SERPINA1, SERPINF1, SERPINF2, TF, TTR, VTN, APOC4	8	40.23076923	AGT, AHSG, ALB, AMBP, APOA1, APOA2, APOA4, APOB, APOC4, APOE, APOH, C3, C4A/C4B, C9, CLU, FETUB, FGA, GC, HPX, ITIH4, KNG1, LCAT, PON1, RBP4, SAA1, SERPINA1, SERPINF1, SERPINF2, TF, TTR, VTN, APOD, APOM, PCYOX1, PLTP	13	D-glucose
Glucocorticoid Receptor Signaling	2.5575	ACTB, AGT, FGG, HSPA8, ICAM1, IL1R2, KRT1, KRT5, KRT76, SCGB1A1, HSPA5, KRT72, KRT79, VCAM1, A2M, KRT14	8	2.778	A2M, ACTB, AGT, FGG, HSPA5, HSPA8, ICAM1, IL1R2, KRT1, KRT5, SCGB1A1, KRT79, HSP90AA1, VCAM1, KRT76, KRT73, KRT14, KRT72	10	1.93025	ACTB, AGT, FGG, HSPA4, HSPA8, KRT1, KRT76, A2M, HSPA5, IL1R2, KRT14, KRT79, SCGB1A1, ICAM1, KRT5, KRT6B	8	2.552846154	ACTB, AGT, FGG, HSPA8, A2M, HSPA5, ICAM1, KRT1, KRT76, SCGB1A1, KRT13, KRT14, KRT79, IL1R2, KRT6B, HSP90AB1, HSPA9, VCAM1, KRT5, KRT72	13	
Gluconeogenesis I	3.56975	ALDOA, ALDOB, BPGM, ENO1, GAPDH, MDH1, PGAM1, FBP1, PGAM2	8	2.7291	ALDOA, ALDOB, BPGM, ENO1, GAPDH, MDH1, PGK1, PGAM2, GPI	10	3.535625	ALDOA, ALDOB, FBP1, PGAM1, PGK1, ENO1, GAPDH, BPGM, MDH1	7	5.114461538	ALDOA, ALDOB, BPGM, ENO1, MDH1, PGAM1, PGAM2, PGK1, ENO3, GAPDH, GPI	13	
Glutathione Redox Reactions I	1.65175	GPX1, GPX3, GSR	8				1.985375	GPX1, GPX3, GSTP1, PRDX6	8	1.824769231	GPX1, GPX3, PRDX6, GSTP1	13	
Glycolysis I	4.5825	ALDOA, ALDOB, BPGM, ENO1, GAPDH, PGAM1, PKM, FBP1, PGAM2	8	3.692	ALDOA, PKM, ALDOB, BPGM, ENO1, GAPDH, PGK1, PGAM2, GPI	10	4.713	ALDOA, ALDOB, FBP1, PGAM1, PGK1, PKM, TPI1, ENO1, GAPDH, BPGM	8	5.69	ALDOA, ALDOB, BPGM, ENO1, PGAM1, PGAM2, PGK1, PKM, ENO3, GAPDH, GPI	13	
GP6 Signaling Pathway	2.56875	COL1A1, COL1A2, FGA, FGB, FGG, COL11A2, COL3A1	8	3.192	COL11A2, COL1A1, COL1A2, COL3A1, FGA, FGB, FGG	10	2.7075	COL1A1, COL1A2, FGA, FGB, FGG, COL3A1, COL11A2	8	2.734615385	COL1A1, COL1A2, FGA, FGB, FGG, COL11A2, COL3A1	13	
Granulocyte Adhesion and Diapedesis	1.56	C5, ICAM1, IL1R2, IL1RAP, SELL, VCAM1, PF4	8	1.6499	C5, ICAM1, IL1R2, IL1RAP, SELL, VCAM1, PF4, MMP2, Ccl8	10				1.578384615	C5, IL1RAP, ICAM1, PF4, SELL, IL1R2, SELP, VCAM1	13	
Growth Hormone Signaling	2.0075	IGF1, IGFALS, IGFBP3, A2M	8	1.908	A2M, IGF1, IGFALS, IGFBP3	10	1.8855	IGFALS, A2M, IGF1, IGFBP3	8	1.924846154	IGFBP3, A2M, IGF1, IGFALS	13	D-glucose
Hematopoiesis from Pluripotent Stem Cells	1.344625	IGHG1, Ighg2a, Ighg2b, IGHM	8										
Hepatic Fibrosis / Hepatic Stellate Cell Activation	7.13	AGT, COL1A1, COL1A2, EGFR, FN1, ICAM1, IFNAR2, IGF1, IGFBP3, IGFBP4, IL1R2, IL1RAP, LBP, VCAM1, A2M, COL11A2, MYH1, COL3A1	8	8.716	A2M, AGT, COL11A2, COL1A1, COL1A2, COL3A1, EGFR, FN1, ICAM1, IFNAR2, IGF1, IGFBP3, IGFBP4, IL1R2, IL1RAP, LBP, FLT4, VCAM1, IGFBP5, MMP2, MYH1	10	6.6875	AGT, COL1A1, COL1A2, EGFR, FN1, IL1RAP, A2M, COL3A1, IFNAR2, IGF1, IGFBP3, IL1R2, LBP, MYH4, ICAM1, IGFBP4, FLT4, COL11A2	8	7.622307692	AGT, COL1A1, COL1A2, EGFR, FN1, IGFBP3, IL1RAP, A2M, COL11A2, ICAM1, IFNAR2, IGF1, LBP, MYH1, IGFBP4, COL3A1, IL1R2, FLT4, VCAM1	13	D-glucose
Hepatic Fibrosis Signaling Pathway				1.7946	AGT, COL11A2, COL1A1, COL1A2, COL3A1, ICAM1, IL1R2, IL1RAP, TF, FLT4, VCAM1, TFRC, BMPR2	10				1.451538462	AGT, COL1A1, COL1A2, IL1RAP, TF, COL11A2, ICAM1, SPP1, COL3A1, IL1R2, TFRC, FLT4, VCAM1	13	

IGF-1 Signaling				1.406	IGF1, IGFBP2, IGFBP3, IGFBP4, IGFBP1, IGFBP5, YWHAZ	10							
IL-12 Signaling and Production in Macrophages	13.0625	ALB, APOA1, APOA2, APOA4, APOB, APOC4, APOD, APOE, APOM, CLU, LYZ, MST1, PCYOX1, PON1, RBP4, SERPINA1, S100A8	8	12.74	ALB, APOA1, APOA2, APOA4, APOB, APOC4, APOD, APOE, APOM, CLU, LYZ, MST1, RBP4, SERPINA1, PON1, PCYOX1	10	12.8875	ALB, APOA1, APOA2, APOA4, APOB, APOD, APOE, APOM, CLU, MST1, PCYOX1, PON1, RBP4, SERPINA1, APOC4, LYZ, S100A8	8	12.96538462	ALB, APOA1, APOA2, APOA4, APOB, APOC4, APOE, CLU, MST1, PON1, RBP4, SERPINA1, APOD, APOM, LYZ, S100A8, PCYOX1	13	D-glucose
IL-6 Signaling	2.04125	COL1A1, CRP, IL1R2, IL1RAP, LBP, A2M	8	2.0229	A2M, COL1A1, CRP, IL1R2, IL1RAP, LBP	10	2.01525	COL1A1, CRP, IL1RAP, A2M, IL1R2, LBP	8	1.929692308	COL1A1, CRP, IL1RAP, A2M, LBP, IL1R2	13	
Intrinsic Prothrombin Activation Pathway	15.05	COL1A1, COL1A2, F12, F13B, F2, FGA, FGB, FGG, KLKB1, KNG1, SERPINC1, F13A1, COL11A2, F11, COL3A1	8	17.23	COL11A2, COL1A1, COL1A2, COL3A1, F12, F13B, F2, FGA, FGB, FGG, KLKB1, KNG1, SERPINC1, F13A1, F5, F10, F9, PROC, PROS1	10	16.925	COL1A1, COL1A2, F10, F12, F13B, F2, F9, FGA, FGB, FGG, KLKB1, KNG1, PROC, PROS1, SERPINC1, COL3A1, F11, F13A1, COL11A2	8	16.36153846	COL1A1, COL1A2, F12, F13A1, F13B, F2, FGA, FGB, FGG, KLKB1, KNG1, SERPINC1, COL11A2, F5, COL3A1, F11	13	
Iron homeostasis signaling pathway	3.46375	CP, EGFR, HBA1/HBA2, Hbb-b1, HP, HPX, TF	8	3.609	CP, EGFR, HBA1/HBA2, Hbb-b1, HP, HPX, TF, TFRC, BMPR2	10	3.51625	CP, EGFR, HBA1/HBA2, Hbb-b1, HP, HPX, TF	8	3.589230769	CP, EGFR, HBA1/HBA2, Hbb-b1, Hbb-b2, HP, HPX, TF, TFRC, HSPA9	13	
Lactose Degradation III				1.402	PSAP	10							
Lipid Antigen Presentation by CD1	1.64475	B2M, PSAP	8	1.769	B2M, PSAP	10	1.6585	B2M, PSAP	8	1.601230769	B2M, PSAP	13	
LPS/IL-1 Mediated Inhibition of RXR Function	2.50125	APOC4, APOE, CAT, FABP4, IL1R2, IL1RAP, PLTP, SOD3, LBP	8	2.359	APOC4, APOE, CAT, IL1R2, IL1RAP, PLTP, SOD3, ALDH1A1, LBP, FABP4	10	3.21375	ALDH1A1, ALDH1L1, APOE, CAT, FABP4, GSTA3, GSTP1, IL1RAP, PLTP, SOD3, APOC4, IL1R2, LBP, FABP5	8	2.588461538	APOC4, APOE, CAT, FABP4, IL1RAP, SOD3, LBP, GSTM5, PLTP, IL1R2, IL4I1, GSTP1	13	
LXR/RXR Activation	45.275	AGT, AHSG, ALB, AMBP, APOA1, APOA2, APOA4, APOB, APOC4, APOD, APOE, APOH, APOM, C3, C4A/C4B, C9, CLU, FGA, GC, HPX, IL1R2, IL1RAP, ITIH4, KNG1, LCAT, LYZ, PCYOX1, PLTP, PON1, RBP4, SAA1, SERPINA1, SERPINF1, SERPINF2, TF, TTR, VTN, LBP, S100A8	8	44.9	AGT, AHSG, ALB, AMBP, APOA1, APOA2, APOA4, APOB, APOC4, APOD, APOE, APOH, APOM, C3, C4A/C4B, C9, CLU, FGA, GC, HPX, IL1R2, IL1RAP, ITIH4, KNG1, LCAT, LYZ, PLTP, RBP4, SAA1, SERPINA1, SERPINF1, SERPINF2, TF, TTR, VTN, LBP, PON1, PCYOX1	10	45.4125	AGT, AHSG, ALB, AMBP, APOA1, APOA2, APOA4, APOB, APOD, APOE, APOH, APOM, C3, C4A/C4B, C9, CLU, FGA, GC, HPX, IL1RAP, ITIH4, KNG1, LCAT, PCYOX1, PLTP, PON1, RBP4, SAA1, SERPINA1, SERPINF1, SERPINF2, TF, TTR, VTN, APOC4, IL1R2, LBP, LYZ, S100A8	8	44.90769231	AGT, AHSG, ALB, AMBP, APOA1, APOA2, APOA4, APOB, APOC4, APOE, APOH, C3, C4A/C4B, C9, CLU, FGA, GC, HPX, IL1RAP, ITIH4, KNG1, LCAT, PON1, RBP4, SAA1, SERPINA1, SERPINF1, SERPINF2, TF, TTR, VTN, APOD, APOM, LBP, LYZ, S100A8, PCYOX1, PLTP, IL1R2	13	
MSP-RON Signaling Pathway	3.40125	ACTB, ACTC1, F12, KLKB1, MST1, F11	8	3.142	ACTA1, ACTB, F12, KLKB1, MST1	10	3.445	ACTB, ACTC1, F12, KLKB1, MST1, F11	8	3.523846154	ACTB, ACTC1, F12, KLKB1, MST1, F11	13	
Neuroprotective Role of THOP1 in Alzheimer's Disease	6.3325	AGT, C1R, CFD, HGFAC, HLA-A, KNG1, PLG, SERPINA3, ACE, FAP, F11	8	6.244	ACE, AGT, C1R, CFD, HGFAC, HLA-A, KNG1, PLG, SERPINA3, FAP, F7	10	6.83125	AGT, C1R, CFD, HGFAC, HLA-A, KNG1, PLG, SERPINA3, ACE, F11, FAP	8	6.575384615	AGT, C1R, CFD, HGFAC, HLA-A, KNG1, PLG, SERPINA3, ACE, FAP, F11	13	
NRF2-mediated Oxidative Stress Response	1.490875	ACTB, ACTC1, CAT, PRDX1, SOD1, SOD3, GSR	8	1.3761	ACTA1, ACTB, CAT, PRDX1, SOD1, SOD3, VCP	10	1.599875	ACTB, ACTC1, CAT, GSTA3, GSTP1, SOD1, SOD3, PRDX1	8	1.624692308	ACTB, ACTC1, CAT, PRDX1, SOD1, SOD3, STIP1, GSTM5, GSTP1	13	
Osteoarthritis Pathway	1.7975	ADIPOQ, FN1, IL1R2, IL1RAP, RBP4, S100A9, S100A8	8	1.681	ADIPOQ, FN1, IL1R2, IL1RAP, RBP4, S100A9, BMPR2	10	1.5575	ADIPOQ, FN1, IL1RAP, RBP4, S100A9, IL1R2, S100A8	8	1.827692308	ADIPOQ, FN1, IL1RAP, RBP4, S100A9, S100A8, SPP1, IL1R2	13	
Phagosome Formation	1.351625	FN1, IGHG1, Ighg2a, Ighg2b, VTN, MBL2	8	1.3472	FN1, Ighg2b, VTN, MBL2, IGHG1, Ighg2a, MRC1	10							
Phagosome Maturation	3.77125	B2M, CTSA, CTSB, CTSZ, HLA-A, LAMP1, LAMP2, PRDX1, PRDX2, CTSE, CTSS	8	3.277	B2M, CTSA, CTSB, HLA-A, LAMP2, PRDX1, PRDX2, CTSE, CTSZ, LAMP1, CTSD	10	2.865	B2M, CTSB, HLA-A, PRDX2, PRDX6, CTSA, LAMP1, PRDX1, LAMP2, CTSE, CTSZ	8	3.473076923	B2M, CTSA, CTSB, HLA-A, PRDX1, PRDX2, PRDX6, CTSE, CTSZ, LAMP1, LAMP2, TUBA1B, TUBB1, TUBB4B	13	citrulline
PPARa/RXRa Activation				1.451	ADIPOQ, APOA1, APOA2, IL1R2, IL1RAP, HSP90AA1, BMPR2	10							
Primary Immunodeficiency Signaling	1.474625	IGHG1, Ighg2a, Ighg2b, IGHM, IL2RG	8										
Production of Nitric Oxide and Reactive Oxygen Species in Macrophages	10.08625	ALB, APOA1, APOA2, APOA4, APOB, APOC4, APOD, APOE, APOM, CAT, CLU, LYZ, PCYOX1, PON1, RBP4, SERPINA1, S100A8	8	9.845	ALB, APOA1, APOA2, APOA4, APOB, APOC4, APOD, APOE, APOM, CAT, CLU, LYZ, RBP4, SERPINA1, PON1, PCYOX1	10	10.1	ALB, APOA1, APOA2, APOA4, APOB, APOD, APOE, APOM, CAT, CLU, PCYOX1, PON1, RBP4, SERPINA1, APOC4, LYZ, S100A8	8	10.11769231	ALB, APOA1, APOA2, APOA4, APOB, APOC4, APOE, CAT, CLU, PON1, RBP4, SERPINA1, APOD, APOM, LYZ, S100A8, PCYOX1	13	
Protein Ubiquitination Pathway	3.412625	B2M, HLA-A, HSPA8, PSMA1, PSMA3, PSMA7, PSMB2, PSMB4, HSPA5, PSMA2, PSMA4, PSMA5, PSMA6, PSMB5, PSMB10, PSMB6, PSMB3, PSMB1	8	3.912	B2M, HLA-A, HSPA5, HSPA8, PSMA1, PSMA3, PSMA7, PSMB2, PSMB4, PSMB6, PSMA2, PSMA4, PSMA5, PSMA6, PSMB3, PSMB5, RPS27A, HSP90AA1, PSMB1, PSMB7	10	3.1825	B2M, HLA-A, HSPA4, HSPA8, PSMA5, PSMA7, PSMB1, HSPA5, PSMA1, PSMA4, PSMA6, PSMB2, PSMB4, PSMB5, PSMA3, PSMB6, PSMA2	8	3.983846154	B2M, HLA-A, HSPA8, PSMA5, PSMA7, PSMB3, PSMB4, HSPA5, PSMA1, PSMA6, PSMB1, PSMB5, PSMB6, PSMA3, PSMB2, PSMA2, PSMA4, HSP90AB1, HSPA9, PSMB10, PSMB7	13	
Regulation of Actin-based Motility by Rho				1.3246	ACTA1, ACTB, ARHGDIA, GSN, CFL1, PFN1	10	1.502875	ACTB, ACTC1, GSN, ARHGDIA, CFL1	8	1.859153846	ACTB, ACTC1, ARHGDIA, GSN, CFL1 ,PFN1	13	
Remodeling of Epithelial Adherens Junctions										1.458615385	ACTB, ACTC1, NME1, VCL, TUBA1B, TUBB1, TUBB4B	13	
Role of IL-17A in Psoriasis	1.66375	S100A9, S100A8	8							1.659230769	S100A9, S100A8	13	
Role of Osteoblasts, Osteoclasts and Chondrocytes in Rheumatoid Arthritis	1.4275	COL1A1, CSF1R, GSN, IGF1, IL1R2, IL1RAP	8	1.6317	COL1A1, CSF1R, GSN, IGF1, IL1R2, IL1RAP, BMPR2	10	1.417125	COL1A1, CSF1R, GSN, IL1RAP, IGF1, IL1R2	8	1.487153846	COL1A1, CSF1R, GSN, IL1RAP, IGF1, SPP1, IL1R2	13	
Role of Pattern Recognition Receptors in Recognition of Bacteria and Viruses	1.354125	C1QB, C1QC, C3, C5, C1QA, MBL2	8	1.5009	C1QB, C3, C5, C1QA, C1QC, MBL2	10							
Role of Tissue Factor in Cancer	2.245	EGFR, F2, FGA, FGB, FGG	8	2.531	EGFR, F2, FGA, FGB, FGG, CFL1, F10, F7	10	2.67125	EGFR, F10, F2, FGA, FGB, FGG, CFL1	8	2.436153846	EGFR, F2, FGA, FGB, FGG, CFL1	13	
SPINK1 Pancreatic Cancer Pathway	4.92625	CPB2, CPN1, CPQ, CTSA, CTSB, KLKB1, CELA1	8	4.919	CPB2, CPN1, CPQ, CTSA, CTSB, KLKB1, CELA1, SPINK1	10	4.56	CPB2, CPN1, CPQ, CTSB, KLKB1, CTSA	8	4.987692308	CPB2, CPN1, CPQ, CTSA, CTSB, KLKB1, CELA1, SPINK1	13	
Sucrose Degradation V (Mammalian)	1.82375	ALDOA, ALDOB	8	1.83	ALDOA, ALDOB	10	1.73125	ALDOA, ALDOB, TPI1	7	1.994615385	ALDOA, ALDOB	13	
Superoxide Radicals Degradation	3.85625	CAT, SOD1, SOD3	8	4.094	CAT, SOD1, SOD3	10	4.295	CAT, SOD1, SOD3	8	4.238461538	CAT, SOD1, SOD3	13	
Systemic Lupus Erythematosus Signaling	3.97125	C5, C8A, C8B, C8G, C9, HLA-A, IGHG1, Ighg2a, Ighg2b, IGHM, KNG1	8	3.681	C5, C8A, C8B, C8G, C9, HLA-A, Ighg2b, IGHM, KNG1, IGHG1, Ighg2a	10	3.57875	C5, C8A, C8B, C8G, C9, HLA-A, KNG1, Ighg2a, IGHM, Ighg2b	8	3.310769231	C5, C8A, C8B, C8G, C9, HLA-A, IGHM, KNG1, IGHG1	13	
Triacylglycerol Degradation										1.346307692	Ces1b/Ces1c, Ces2e, PRDX6	13	
Virus Entry via Endocytic Pathways	1.47275	ACTB, ACTC1, B2M, HLA-A	8	1.5243	ACTA1, ACTB, B2M, HLA-A, ITGB4, TFRC	10	1.4405	ACTB, ACTC1, B2M, HLA-A	8	1.491076923	ACTB, ACTC1, B2M, HLA-A, TFRC	13	

Supplementary Table 2: Protein abundances were input into Ingenuity Pathway Analysis (IPA®, Qiagen) and mapped against the mouse Ingenuity Knowledgebase. This revealed 83 unique enriched pathways, with 41 (49.4%) shared across all conditions. Though pathways unique to sham, TBI, sham RIC, and TBI RIC were identified, no pathways were shared only between the two RIC groups and one pathway was uniquely enriched in the two TBI groups.

Accession	Description	Gene	ID	pVal	pVal.Adj
P68134	Actin, alpha skeletal muscle OS=Mus musculus GN=Acta1 PE=1 SV=1	Acta1	P68134	3.71517E-06	0.001805574
Q922U2	Keratin, type II cytoskeletal 5 OS=Mus musculus GN=Krt5 PE=1 SV=1	Krt5	Q922U2	0.000451923	0.109817343
Q3V1H1	Cytoskeleton-associated protein 2 OS=Mus musculus GN=Ckap2 PE=1 SV=1	Ckap2	Q3V1H1	0.0007891	0.12783426
P68033	Actin, alpha cardiac muscle 1 OS=Mus musculus GN=Actc1 PE=1 SV=1	Actc1	P68033	0.0017875	0.187283659
Q3UAW9	Transcription factor IIIB 50 kDa subunit OS=Mus musculus GN=Brf2 PE=1 SV=1	Brf2	Q3UAW9	0.001926787	0.187283659
Q9DA19	Corepressor interacting with RBPJ 1 OS=Mus musculus GN=Cir1 PE=1 SV=2	Cir1	Q9DA19	0.002784608	0.225553282
O08576	RUN domain-containing protein 3A OS=Mus musculus GN=Rundc3a PE=1 SV=1	Rundc3a	O08576	0.00658146	0.29454115
P01942	Hemoglobin subunit alpha OS=Mus musculus GN=Hba PE=1 SV=2	Hba	P01942	0.007878673	0.29454115
P04117	Fatty acid-binding protein, adipocyte OS=Mus musculus GN=Fabp4 PE=1 SV=3	Fabp4	P04117	0.005085734	0.29454115
Q08879-2	Isoform C of Fibulin-1 OS=Mus musculus GN=Fbln1	Fbln1	Q08879-2	0.00691215	0.29454115
Q5SX39	Myosin-4 OS=Mus musculus GN=Myh4 PE=2 SV=1	Myh4	Q5SX39	0.00658146	0.29454115
Q61646	Haptoglobin OS=Mus musculus GN=Hp PE=1 SV=1	Hp	Q61646	0.007844275	0.29454115
Q8VDJ3	Vigilin OS=Mus musculus GN=Hdlbp PE=1 SV=1	Hdlbp	Q8VDJ3	0.00658146	0.29454115
Q9JM99-3	Isoform C of Proteoglycan 4 OS=Mus musculus GN=Prg4	Prg4	Q9JM99-3	0.01229414	0.426782278
O09049	Regenerating islet-derived protein 3-gamma OS=Mus musculus GN=Reg3g PE=1 SV=1	Reg3g	O09049	0.016422713	0.532095911
P17563	Selenium-binding protein 1 OS=Mus musculus GN=Selenbp1 PE=1 SV=2	Selenbp1	P17563	0.020235805	0.578505954
P63017	Heat shock cognate 71 kDa protein OS=Mus musculus GN=Hspa8 PE=1 SV=1	Hspa8	P63017	0.020034504	0.578505954
A2ARV4	Low-density lipoprotein receptor-related protein 2 OS=Mus musculus GN=Lrp2 PE=1 SV=1	Lrp2	A2ARV4	0.275282071	0.752153761
B2RXS4	Plexin-B2 OS=Mus musculus GN=Plxnb2 PE=1 SV=1	Plxnb2	B2RXS4	0.275282071	0.752153761
B5X0G2	Major urinary protein 17 OS=Mus musculus GN=Mup17 PE=2 SV=2	Mup17	B5X0G2	0.170019218	0.752153761
G5E8Q8	Adhesion G protein-coupled receptor F5 OS=Mus musculus GN=Adgrf5 PE=1 SV=1	Adgrf5	G5E8Q8	0.225674194	0.752153761
O08689	Growth/differentiation factor 8 OS=Mus musculus GN=Mstn PE=1 SV=1	Mstn	O08689	0.275282071	0.752153761
O08709	Peroxiredoxin-6 OS=Mus musculus GN=Prdx6 PE=1 SV=3	Prdx6	O08709	0.109883878	0.752153761
O08742	Platelet glycoprotein V OS=Mus musculus GN=Gp5 PE=1 SV=1	Gp5	O08742	0.275282071	0.752153761
O09043	Napsin-A OS=Mus musculus GN=Napsa PE=1 SV=1	Napsa	O09043	0.225895457	0.752153761
O09051	Guanylate cyclase activator 2B OS=Mus musculus GN=Guca2b PE=1 SV=2	Guca2b	O09051	0.275282071	0.752153761
O09061	Proteasome subunit beta type-1 OS=Mus musculus GN=Psmb1 PE=1 SV=1	Psmb1	O09061	0.097632074	0.752153761
O09159	Lysosomal alpha-mannosidase OS=Mus musculus GN=Man2b1 PE=1 SV=4	Man2b1	O09159	0.276018142	0.752153761
O35526	Syntaxin-1A OS=Mus musculus GN=Stx1a PE=1 SV=3	Stx1a	O35526	0.275282071	0.752153761
O35744	Chitinase-like protein 3 OS=Mus musculus GN=Chil3 PE=1 SV=2	Chil3	O35744	0.114456007	0.752153761
O35955	Proteasome subunit beta type-10 OS=Mus musculus GN=Psmb10 PE=1 SV=1	Psmb10	O35955	0.191775298	0.752153761
O70362	Phosphatidylinositol-glycan-specific phospholipase D OS=Mus musculus GN=Gpld1 PE=1 SV=1	Gpld1	O70362	0.057316827	0.752153761
O70370	Cathepsin S OS=Mus musculus GN=Ctss PE=1 SV=2	Ctss	O70370	0.275282071	0.752153761
O70435	Proteasome subunit alpha type-3 OS=Mus musculus GN=Psma3 PE=1 SV=3	Psma3	O70435	0.284461479	0.752153761
O88783	Coagulation factor V OS=Mus musculus GN=F5 PE=1 SV=1	F5	O88783	0.178478816	0.752153761
O88947	Coagulation factor X OS=Mus musculus GN=F10 PE=1 SV=1	F10	O88947	0.057313381	0.752153761
O88968	Transcobalamin-2 OS=Mus musculus GN=Tcn2 PE=1 SV=1	Tcn2	O88968	0.23694453	0.752153761
O89020	Afamin OS=Mus musculus GN=Afm PE=1 SV=2	Afm	O89020	0.176146122	0.752153761
P00329	Alcohol dehydrogenase 1 OS=Mus musculus GN=Adh1 PE=1 SV=2	Adh1	P00329	0.275282071	0.752153761
P00687	Alpha-amylase 1 OS=Mus musculus GN=Amy1 PE=1 SV=2	Amy1	P00687	0.158337287	0.752153761
P00688	Pancreatic alpha-amylase OS=Mus musculus GN=Amy2 PE=1 SV=2	Amy2	P00688	0.211832237	0.752153761
P00920	Carbonic anhydrase 2 OS=Mus musculus GN=Ca2 PE=1 SV=4	Ca2	P00920	0.068232018	0.752153761
P01029	Complement C4-B OS=Mus musculus GN=C4b PE=1 SV=3	C4b	P01029	0.191201341	0.752153761
P01726	Ig lambda-1 chain V region H2020 OS=Mus musculus PE=3 SV=1	0	P01726	0.10107327	0.752153761
P01843	Ig lambda-1 chain C region OS=Mus musculus PE=1 SV=1	0	P01843	0.218468895	0.752153761
P01867	Ig gamma-2B chain C region OS=Mus musculus GN=Igh-3 PE=1 SV=3	Igh-3	P01867	0.25840777	0.752153761
P01869	Ig gamma-1 chain C region, membrane-bound form OS=Mus musculus GN=Ighg1 PE=1 SV=2	Ighg1	P01869	0.032296669	0.752153761
P01878	Ig alpha chain C region OS=Mus musculus PE=1 SV=1	0	P01878	0.260902406	0.752153761
P01887	Beta-2-microglobulin OS=Mus musculus GN=B2m PE=1 SV=2	B2m	P01887	0.288866698	0.752153761
P02088	Hemoglobin subunit beta-1 OS=Mus musculus GN=Hbb-b1 PE=1 SV=2	Hbb-b1	P02088	0.071524811	0.752153761
P04104	Keratin, type II cytoskeletal 1 OS=Mus musculus GN=Krt1 PE=1 SV=4	Krt1	P04104	0.198771176	0.752153761
P04186	Complement factor B OS=Mus musculus GN=Cfb PE=1 SV=2	Cfb	P04186	0.15480166	0.752153761
P04939	Major urinary protein 3 OS=Mus musculus GN=Mup3 PE=1 SV=1	Mup3	P04939	0.194903962	0.752153761
P06151	L-lactate dehydrogenase A chain OS=Mus musculus GN=Ldha PE=1 SV=3	Ldha	P06151	0.16812035	0.752153761
P07758	Alpha-1-antitrypsin 1-1 OS=Mus musculus GN=Serpina1a PE=1 SV=4	Serpina1a	P07758	0.280386931	0.752153761
P07759	Serine protease inhibitor A3K OS=Mus musculus GN=Serpina3k PE=1 SV=2	Serpina3k	P07759	0.182326655	0.752153761
P08121	Collagen alpha-1(III) chain OS=Mus musculus GN=Col3a1 PE=1 SV=4	Col3a1	P08121	0.163491643	0.752153761
P08226	Apolipoprotein E OS=Mus musculus GN=Apoe PE=1 SV=2	Apoe	P08226	0.085158663	0.752153761
P08228	Superoxide dismutase [Cu-Zn] OS=Mus musculus GN=Sod1 PE=1 SV=2	Sod1	P08228	0.111572087	0.752153761
P09581	Macrophage colony-stimulating factor 1 receptor OS=Mus musculus GN=Csf1r PE=1 SV=3	Csf1r	P09581	0.289408957	0.752153761
P10605	Cathepsin B OS=Mus musculus GN=Ctsb PE=1 SV=2	Ctsb	P10605	0.065607569	0.752153761
P10923	Osteopontin OS=Mus musculus GN=Spp1 PE=1 SV=1	Spp1	P10923	0.096981616	0.752153761
P11352	Glutathione peroxidase 1 OS=Mus musculus GN=Gpx1 PE=1 SV=2	Gpx1	P11352	0.272483207	0.752153761
P12804	Fibroleukin OS=Mus musculus GN=Fgl2 PE=1 SV=1	Fgl2	P12804	0.275282071	0.752153761
P14152	Malate dehydrogenase, cytoplasmic OS=Mus musculus GN=Mdh1 PE=1 SV=3	Mdh1	P14152	0.174702203	0.752153761
P14428	H-2 class I histocompatibility antigen, K-Q alpha chain (Fragment) OS=Mus musculus GN=H2-K1 PE=1 SV=1	H2-K1	P14428	0.275282071	0.752153761
P15532	Nucleoside diphosphate kinase A OS=Mus musculus GN=Nme1 PE=1 SV=1	Nme1	P15532	0.241540437	0.752153761
P16015	Carbonic anhydrase 3 OS=Mus musculus GN=Ca3 PE=1 SV=3	Ca3	P16015	0.275282071	0.752153761
P17751	Triosephosphate isomerase OS=Mus musculus GN=Tpi1 PE=1 SV=4	Tpi1	P17751	0.275282071	0.752153761
P17897	Lysozyme C-1 OS=Mus musculus GN=Lyz1 PE=1 SV=1	Lyz1	P17897	0.172428236	0.752153761
P18760	Cofilin-1 OS=Mus musculus GN=Cfl1 PE=1 SV=3	Cfl1	P18760	0.285018298	0.752153761
P20065	Thymosin beta-4 OS=Mus musculus GN=Tmsb4x PE=1 SV=1	Tmsb4x	P20065	0.225895457	0.752153761
P21460	Cystatin-C OS=Mus musculus GN=Cst3 PE=1 SV=2	Cst3	P21460	0.170956015	0.752153761
P21550	Beta-enolase OS=Mus musculus GN=Eno3 PE=1 SV=3	Eno3	P21550	0.096981616	0.752153761
P24270	Catalase OS=Mus musculus GN=Cat PE=1 SV=4	Cat	P24270	0.207666061	0.752153761
P26262	Plasma kallikrein OS=Mus musculus GN=Klkb1 PE=1 SV=2	Klkb1	P26262	0.060579308	0.752153761
P27005	Protein S100-A8 OS=Mus musculus GN=S100a8 PE=1 SV=3	S100a8	P27005	0.116346306	0.752153761
P28650-2	Isoform 2 of Adenylosuccinate synthetase isozyme 1 OS=Mus musculus GN=Adssl1	Adssl1	P28650-2	0.096981616	0.752153761
P28665	Murinoglobulin-1 OS=Mus musculus GN=Mug1 PE=1 SV=3	Mug1	P28665	0.286941216	0.752153761
P28798	Granulins OS=Mus musculus GN=Grn PE=1 SV=2	Grn	P28798	0.126984492	0.752153761
P29533	Vascular cell adhesion protein 1 OS=Mus musculus GN=Vcam1 PE=1 SV=1	Vcam1	P29533	0.222163877	0.752153761
P30115	Glutathione S-transferase A3 OS=Mus musculus GN=Gsta3 PE=1 SV=2	Gsta3	P30115	0.275282071	0.752153761

P31725	Protein S100-A9 OS=Mus musculus GN=S100a9 PE=1 SV=3	S100a9	P31725	0.141631463	0.752153761
P31786	Acyl-CoA-binding protein OS=Mus musculus GN=Dbi PE=1 SV=2	Dbi	P31786	0.096981616	0.752153761
P32848	Parvalbumin alpha OS=Mus musculus GN=Pvalb PE=1 SV=3	Pvalb	P32848	0.0882279	0.752153761
P34884	Macrophage migration inhibitory factor OS=Mus musculus GN=Mif PE=1 SV=2	Mif	P34884	0.046973125	0.752153761
P35441	Thrombospondin-1 OS=Mus musculus GN=Thbs1 PE=1 SV=1	Thbs1	P35441	0.159411765	0.752153761
P35455	Vasopressin-neurophysin 2-copeptin OS=Mus musculus GN=Avp PE=2 SV=1	Avp	P35455	0.096981616	0.752153761
P35917	Vascular endothelial growth factor receptor 3 OS=Mus musculus GN=Flt4 PE=1 SV=1	Flt4	P35917	0.253842026	0.752153761
P39039	Mannose-binding protein A OS=Mus musculus GN=Mbl1 PE=1 SV=1	Mbl1	P39039	0.107264873	0.752153761
P39447	Tight junction protein ZO-1 OS=Mus musculus GN=Tjp1 PE=1 SV=2	Tjp1	P39447	0.250319656	0.752153761
P42703	Leukemia inhibitory factor receptor OS=Mus musculus GN=Lifr PE=1 SV=1	Lifr	P42703	0.116110084	0.752153761
P45700	Mannosyl-oligosaccharide 1,2-alpha-mannosidase IA OS=Mus musculus GN=Man1a1 PE=1 SV=1	Man1a1	P45700	0.225356415	0.752153761
P46412	Glutathione peroxidase 3 OS=Mus musculus GN=Gpx3 PE=1 SV=2	Gpx3	P46412	0.159419194	0.752153761
P47791	Glutathione reductase, mitochondrial OS=Mus musculus GN=Gsr PE=1 SV=3	Gsr	P47791	0.046973125	0.752153761
P48301	Transcriptional enhancer factor TEF-4 OS=Mus musculus GN=Tead2 PE=2 SV=1	Tead2	P48301	0.275282071	0.752153761
P49722	Proteasome subunit alpha type-2 OS=Mus musculus GN=Psma2 PE=1 SV=3	Psma2	P49722	0.079985381	0.752153761
P50247	Adenosylhomocysteinase OS=Mus musculus GN=Ahcy PE=1 SV=3	Ahcy	P50247	0.275282071	0.752153761
P51885	Lumican OS=Mus musculus GN=Lum PE=1 SV=2	Lum	P51885	0.036187359	0.752153761
P60710	Actin, cytoplasmic 1 OS=Mus musculus GN=Actb PE=1 SV=1	Actb	P60710	0.21206075	0.752153761
P62827	GTP-binding nuclear protein Ran OS=Mus musculus GN=Ran PE=1 SV=3	Ran	P62827	0.275282071	0.752153761
P63242	Eukaryotic translation initiation factor 5A-1 OS=Mus musculus GN=Eif5a PE=1 SV=2	Eif5a	P63242	0.275282071	0.752153761
P70274	Selenoprotein P OS=Mus musculus GN=Sepp1 PE=1 SV=3	Sepp1	P70274	0.109227467	0.752153761
P97290	Plasma protease C1 inhibitor OS=Mus musculus GN=Serping1 PE=1 SV=3	Serping1	P97290	0.17859037	0.752153761
P97300	Neuroplastin OS=Mus musculus GN=Nptn PE=1 SV=3	Nptn	P97300	0.059723215	0.752153761
P97443	Histone-lysine N-methyltransferase Smyd1 OS=Mus musculus GN=Smyd1 PE=1 SV=3	Smyd1	P97443	0.275282071	0.752153761
P97445	Voltage-dependent P/Q-type calcium channel subunit alpha-1A OS=Mus musculus GN=Cacna1a PE=1 SV=2	Cacna1a	P97445	0.275282071	0.752153761
P97449	Aminopeptidase N OS=Mus musculus GN=Anpep PE=1 SV=4	Anpep	P97449	0.07742397	0.752153761
P98086	Complement C1q subcomponent subunit A OS=Mus musculus GN=C1qa PE=1 SV=2	C1qa	P98086	0.161367024	0.752153761
Q00623	Apolipoprotein A-I OS=Mus musculus GN=Apoa1 PE=1 SV=2	Apoa1	Q00623	0.285098076	0.752153761
Q01279	Epidermal growth factor receptor OS=Mus musculus GN=Egfr PE=1 SV=1	Egfr	Q01279	0.212775053	0.752153761
Q02819	Nucleobindin-1 OS=Mus musculus GN=Nucb1 PE=1 SV=2	Nucb1	Q02819	0.275282071	0.752153761
Q03401	Cysteine-rich secretory protein 1 OS=Mus musculus GN=Crisp1 PE=2 SV=1	Crisp1	Q03401	0.275282071	0.752153761
Q04447	Creatine kinase B-type OS=Mus musculus GN=Ckb PE=1 SV=1	Ckb	Q04447	0.275282071	0.752153761
Q05816	Fatty acid-binding protein, epidermal OS=Mus musculus GN=Fabp5 PE=1 SV=3	Fabp5	Q05816	0.275282071	0.752153761
Q06318	Uteroglobin OS=Mus musculus GN=Scgb1a1 PE=1 SV=1	Scgb1a1	Q06318	0.042860069	0.752153761
Q06890	Clusterin OS=Mus musculus GN=Clu PE=1 SV=1	Clu	Q06890	0.284985824	0.752153761
Q07456	Protein AMBP OS=Mus musculus GN=Ambp PE=1 SV=2	Ambp	Q07456	0.161047475	0.752153761
Q08481	Platelet endothelial cell adhesion molecule OS=Mus musculus GN=Pecam1 PE=1 SV=1	Pecam1	Q08481	0.275282071	0.752153761
Q0VF94	NTPase KAP family P-loop domain-containing protein 1 OS=Mus musculus GN=Nkpd1 PE=2 SV=2	Nkpd1	Q0VF94	0.275282071	0.752153761
Q3UV17	Keratin, type II cytoskeletal 2 oral OS=Mus musculus GN=Krt76 PE=1 SV=1	Krt76	Q3UV17	0.200592007	0.752153761
Q3V0Q1	Dynein heavy chain 12, axonemal OS=Mus musculus GN=Dnah12 PE=1 SV=2	Dnah12	Q3V0Q1	0.144776802	0.752153761
Q4VAA2	Protein CDV3 OS=Mus musculus GN=Cdv3 PE=1 SV=2	Cdv3	Q4VAA2	0.034262829	0.752153761
Q5SSW2	Proteasome activator complex subunit 4 OS=Mus musculus GN=Psme4 PE=1 SV=1	Psme4	Q5SSW2	0.182770207	0.752153761
Q5SX40	Myosin-1 OS=Mus musculus GN=Myh1 PE=1 SV=1	Myh1	Q5SX40	0.160012826	0.752153761
Q60864	Stress-induced-phosphoprotein 1 OS=Mus musculus GN=Stip1 PE=1 SV=1	Stip1	Q60864	0.096981616	0.752153761
Q60963	Platelet-activating factor acetylhydrolase OS=Mus musculus GN=Pla2g7 PE=2 SV=2	Pla2g7	Q60963	0.031345529	0.752153761
Q61147	Ceruloplasmin OS=Mus musculus GN=Cp PE=1 SV=2	Cp	Q61147	0.058774892	0.752153761
Q61171	Peroxiredoxin-2 OS=Mus musculus GN=Prdx2 PE=1 SV=3	Prdx2	Q61171	0.052170623	0.752153761
Q61206	Platelet-activating factor acetylhydrolase IB subunit beta OS=Mus musculus GN=Pafah1b2 PE=1 SV=2	Pafah1b2	Q61206	0.275282071	0.752153761
Q61207	Prosaposin OS=Mus musculus GN=Psap PE=1 SV=2	Psap	Q61207	0.246383769	0.752153761
Q61247	Alpha-2-antiplasmin OS=Mus musculus GN=Serpinf2 PE=1 SV=1	Serpinf2	Q61247	0.127862389	0.752153761
Q61316	Heat shock 70 kDa protein 4 OS=Mus musculus GN=Hspa4 PE=1 SV=1	Hspa4	Q61316	0.275282071	0.752153761
Q61703	Inter-alpha-trypsin inhibitor heavy chain H2 OS=Mus musculus GN=Itih2 PE=1 SV=1	Itih2	Q61703	0.273541486	0.752153761
Q62209	Synaptonemal complex protein 1 OS=Mus musculus GN=Sycp1 PE=1 SV=2	Sycp1	Q62209	0.275282071	0.752153761
Q64695	Endothelial protein C receptor OS=Mus musculus GN=Procr PE=1 SV=3	Procr	Q64695	0.275282071	0.752153761
Q64727	Vinculin OS=Mus musculus GN=Vcl PE=1 SV=4	Vcl	Q64727	0.250319656	0.752153761
Q64739	Collagen alpha-2(XI) chain OS=Mus musculus GN=Col11a2 PE=2 SV=3	Col11a2	Q64739	0.107585767	0.752153761
Q6NXH9	Keratin, type II cytoskeletal 73 OS=Mus musculus GN=Krt73 PE=1 SV=1	Krt73	Q6NXH9	0.113937682	0.752153761
Q6P253-4	Isoform 4 of Dermokine OS=Mus musculus GN=Dmkn	Dmkn	Q6P253-4	0.268835268	0.752153761
Q6PDM2	Serine/arginine-rich splicing factor 1 OS=Mus musculus GN=Srsf1 PE=1 SV=3	Srsf1	Q6PDM2	0.275282071	0.752153761
Q6PI62	Probable G-protein coupled receptor 173 OS=Mus musculus GN=Gpr173 PE=2 SV=1	Gpr173	Q6PI62	0.275282071	0.752153761
Q7TNS2	MICOS complex subunit Mic10 OS=Mus musculus GN=Minos1 PE=1 SV=1	Minos1	Q7TNS2	0.275282071	0.752153761
Q80U93	Nuclear pore complex protein Nup214 OS=Mus musculus GN=Nup214 PE=1 SV=2	Nup214	Q80U93	0.275282071	0.752153761
Q80XP9	Serine/threonine-protein kinase WNK3 OS=Mus musculus GN=Wnk3 PE=1 SV=3	Wnk3	Q80XP9	0.275282071	0.752153761
Q8BI17	SH2 domain-containing protein 7 OS=Mus musculus GN=Sh2d7 PE=2 SV=2	Sh2d7	Q8BI17	0.275282071	0.752153761
Q8BND5	Sulfhydryl oxidase 1 OS=Mus musculus GN=Qsox1 PE=1 SV=1	Qsox1	Q8BND5	0.081047076	0.752153761
Q8BYH3	tRNA:m(4)X modification enzyme TRM13 homolog OS=Mus musculus GN=Trmt13 PE=2 SV=1	Trmt13	Q8BYH3	0.275282071	0.752153761
Q8CFG8	Complement C1s-B subcomponent OS=Mus musculus GN=C1sb PE=2 SV=1	C1sb	Q8CFG8	0.275282071	0.752153761
Q8CG65	SCO-spondin OS=Mus musculus GN=Sspo PE=2 SV=2	Sspo	Q8CG65	0.275282071	0.752153761
Q8CIT9	Suprabasin OS=Mus musculus GN=Sbsn PE=2 SV=1	Sbsn	Q8CIT9	0.275282071	0.752153761
Q8K182	Complement component C8 alpha chain OS=Mus musculus GN=C8a PE=1 SV=1	C8a	Q8K182	0.050936138	0.752153761
Q8K389	CDK5 regulatory subunit-associated protein 2 OS=Mus musculus GN=Cdk5rap2 PE=1 SV=3	Cdk5rap2	Q8K389	0.250319656	0.752153761
Q8K4X7	1-acyl-sn-glycerol-3-phosphate acyltransferase delta OS=Mus musculus GN=Agpat4 PE=1 SV=1	Agpat4	Q8K4X7	0.275282071	0.752153761
Q8R0Y6	Cytosolic 10-formyltetrahydrofolate dehydrogenase OS=Mus musculus GN=Aldh1l1 PE=1 SV=1	Aldh1l1	Q8R0Y6	0.275282071	0.752153761
Q8R146	Acylamino-acid-releasing enzyme OS=Mus musculus GN=Apeh PE=1 SV=3	Apeh	Q8R146	0.275282071	0.752153761
Q8R242	Di-N-acetylchitobiase OS=Mus musculus GN=Ctbs PE=1 SV=2	Ctbs	Q8R242	0.125207729	0.752153761
Q8VCT4	Carboxylesterase 1D OS=Mus musculus GN=Ces1d PE=1 SV=1	Ces1d	Q8VCT4	0.275282071	0.752153761
Q91Y47	Coagulation factor XI OS=Mus musculus GN=F11 PE=2 SV=2	F11	Q91Y47	0.26708923	0.752153761
Q99LX0	Protein deglycase DJ-1 OS=Mus musculus GN=Park7 PE=1 SV=1	Park7	Q99LX0	0.250319656	0.752153761
Q99PT1	Rho GDP-dissociation inhibitor 1 OS=Mus musculus GN=Arhgdia PE=1 SV=3	Arhgdia	Q99PT1	0.051114795	0.752153761
Q99PT9	Kinesin-like protein KIF19 OS=Mus musculus GN=Kif19 PE=1 SV=2	Kif19	Q99PT9	0.275282071	0.752153761
Q9CPX6	Ubiquitin-like-conjugating enzyme ATG3 OS=Mus musculus GN=Atg3 PE=1 SV=1	Atg3	Q9CPX6	0.275282071	0.752153761
Q9CR86	Calcium-regulated heat stable protein 1 OS=Mus musculus GN=Carhsp1 PE=1 SV=1	Carhsp1	Q9CR86	0.250319656	0.752153761
Q9CWU0	Putative ATP-dependent RNA helicase TDRD12 OS=Mus musculus GN=Tdrd12 PE=1 SV=2	Tdrd12	Q9CWU0	0.275282071	0.752153761

Q9CZT5	Vasorin OS=Mus musculus GN=Vasn PE=2 SV=2	Vasn	Q9CZT5	0.225895457	0.752153761
Q9DBB9	Carboxypeptidase N subunit 2 OS=Mus musculus GN=Cpn2 PE=1 SV=2	Cpn2	Q9DBB9	0.233017739	0.752153761
Q9DBG7	Signal recognition particle receptor subunit alpha OS=Mus musculus GN=Srpr PE=1 SV=1	Srpr	Q9DBG7	0.276441396	0.752153761
Q9EPL0	Xylosyltransferase 2 OS=Mus musculus GN=Xylt2 PE=2 SV=3	Xylt2	Q9EPL0	0.275282071	0.752153761
Q9EPW0	Type I inositol 3,4-bisphosphate 4-phosphatase OS=Mus musculus GN=Inpp4a PE=1 SV=1	Inpp4a	Q9EPW0	0.275282071	0.752153761
Q9EQZ6-2	Isoform 2 of Rap guanine nucleotide exchange factor 4 OS=Mus musculus GN=Rapgef4	Rapgef4	Q9EQZ6-2	0.275282071	0.752153761
Q9JHH6	Carboxypeptidase B2 OS=Mus musculus GN=Cpb2 PE=1 SV=1	Cpb2	Q9JHH6	0.113985327	0.752153761
Q9JI02	Secretoglobin family 2B member 20 OS=Mus musculus GN=Scgb2b20 PE=2 SV=1	Scgb2b20	Q9JI02	0.201867004	0.752153761
Q9JI78	Peptide-N(4)-(N-acetyl-beta-glucosaminyl)asparagine amidase OS=Mus musculus GN=Ngly1 PE=1 SV=2	Ngly1	Q9JI78	0.275282071	0.752153761
Q9JIZ0	Probable N-acetyltransferase CML1 OS=Mus musculus GN=Cml1 PE=1 SV=1	Cml1	Q9JIZ0	0.275282071	0.752153761
Q9QUM9	Proteasome subunit alpha type-6 OS=Mus musculus GN=Psma6 PE=1 SV=1	Psma6	Q9QUM9	0.075561135	0.752153761
Q9QXD6	Fructose-1,6-bisphosphatase 1 OS=Mus musculus GN=Fbp1 PE=1 SV=3	Fbp1	Q9QXD6	0.133696228	0.752153761
Q9QXF8	Glycine N-methyltransferase OS=Mus musculus GN=Gnmt PE=1 SV=3	Gnmt	Q9QXF8	0.275282071	0.752153761
Q9QXL8	Nucleoside diphosphate kinase 7 OS=Mus musculus GN=Nme7 PE=1 SV=1	Nme7	Q9QXL8	0.275282071	0.752153761
Q9R098	Hepatocyte growth factor activator OS=Mus musculus GN=Hgfac PE=1 SV=1	Hgfac	Q9R098	0.205585767	0.752153761
Q9R111	Guanine deaminase OS=Mus musculus GN=Gda PE=1 SV=1	Gda	Q9R111	0.112246237	0.752153761
Q9R182	Angiopoietin-related protein 3 OS=Mus musculus GN=Angptl3 PE=2 SV=1	Angptl3	Q9R182	0.284741937	0.752153761
Q9WUB3	Glycogen phosphorylase, muscle form OS=Mus musculus GN=Pygm PE=1 SV=3	Pygm	Q9WUB3	0.156018785	0.752153761
Q9WUU7	Cathepsin Z OS=Mus musculus GN=Ctsz PE=1 SV=1	Ctsz	Q9WUU7	0.131303585	0.752153761
Q9WVM6	Tolloid-like protein 2 OS=Mus musculus GN=Tll2 PE=1 SV=1	Tll2	Q9WVM6	0.230703519	0.752153761
Q9Z0M9	Interleukin-18-binding protein OS=Mus musculus GN=Il18bp PE=1 SV=2	Il18bp	Q9Z0M9	0.213875833	0.752153761
Q9Z1T2	Thrombospondin-4 OS=Mus musculus GN=Thbs4 PE=1 SV=1	Thbs4	Q9Z1T2	0.160081132	0.752153761
P01795	Ig heavy chain V region M167 OS=Mus musculus PE=1 SV=1	0	P01795	0.294439153	0.761156534
A2A863	Integrin beta-4 OS=Mus musculus GN=Itgb4 PE=1 SV=1	Itgb4	A2A863	0.407301567	0.785750244
A2AQ07	Tubulin beta-1 chain OS=Mus musculus GN=Tubb1 PE=1 SV=1	Tubb1	A2AQ07	0.572406704	0.785750244
A6H6A4	Leucine-rich repeat and IQ domain-containing protein 4 OS=Mus musculus GN=Lrriq4 PE=2 SV=1	Lrriq4	A6H6A4	0.407301567	0.785750244
A6X935	Inter alpha-trypsin inhibitor, heavy chain 4 OS=Mus musculus GN=Itih4 PE=1 SV=2	Itih4	A6X935	0.435339957	0.785750244
B2RUR8	OTU domain-containing protein 7B OS=Mus musculus GN=Otud7b PE=1 SV=1	Otud7b	B2RUR8	0.407301567	0.785750244
D3Z750	Maestro heat-like repeat-containing protein family member 2A OS=Mus musculus GN=Mroh2a PE=1 SV=2	Mroh2a	D3Z750	0.572406704	0.785750244
O08677	Kininogen-1 OS=Mus musculus GN=Kng1 PE=1 SV=1	Kng1	O08677	0.320963268	0.785750244
O09046-2	Isoform 2 of L-amino-acid oxidase OS=Mus musculus GN=Il4i1	Il4i1	O09046-2	0.572406704	0.785750244
O09164	Extracellular superoxide dismutase [Cu-Zn] OS=Mus musculus GN=Sod3 PE=1 SV=1	Sod3	O09164	0.431950518	0.785750244
O35607	Bone morphogenetic protein receptor type-2 OS=Mus musculus GN=Bmpr2 PE=1 SV=1	Bmpr2	O35607	0.407301567	0.785750244
O35658	Complement component 1 Q subcomponent-binding protein, mitochondrial OS=Mus musculus GN=C1qbp PE=1 SV=1	C1qbp	O35658	0.572406704	0.785750244
O35664-3	Isoform 3 of Interferon alpha/beta receptor 2 OS=Mus musculus GN=Ifnar2	Ifnar2	O35664-3	0.538653338	0.785750244
O54819	Tissue factor pathway inhibitor OS=Mus musculus GN=Tfpi PE=2 SV=1	Tfpi	O54819	0.407301567	0.785750244
O70165	Ficolin-1 OS=Mus musculus GN=Fcn1 PE=1 SV=1	Fcn1	O70165	0.522637485	0.785750244
P01592	Immunoglobulin J chain OS=Mus musculus GN=Jchain PE=1 SV=4	Jchain	P01592	0.447900681	0.785750244
P01751	Ig heavy chain V region B1-8/186-2 OS=Mus musculus GN=Ighv1-72 PE=1 SV=1	Ighv1-72	P01751	0.572406704	0.785750244
P01865	Ig gamma-2A chain C region, membrane-bound form OS=Mus musculus GN=Igh-1a PE=1 SV=3	Igh-1a	P01865	0.36842453	0.785750244
P01872	Ig mu chain C region OS=Mus musculus GN=Ighm PE=1 SV=2	Ighm	P01872	0.563754203	0.785750244
P02089	Hemoglobin subunit beta-2 OS=Mus musculus GN=Hbb-b2 PE=1 SV=2	Hbb-b2	P02089	0.572406704	0.785750244
P04247	Myoglobin OS=Mus musculus GN=Mb PE=1 SV=3	Mb	P04247	0.572406704	0.785750244
P05213	Tubulin alpha-1B chain OS=Mus musculus GN=Tuba1b PE=1 SV=2	Tuba1b	P05213	0.572406704	0.785750244
P05366	Serum amyloid A-1 protein OS=Mus musculus GN=Saa1 PE=1 SV=2	Saa1	P05366	0.550387993	0.785750244
P06683	Complement component C9 OS=Mus musculus GN=C9 PE=1 SV=2	C9	P06683	0.381892424	0.785750244
P06728	Apolipoprotein A-IV OS=Mus musculus GN=Apoa4 PE=1 SV=3	Apoa4	P06728	0.495918754	0.785750244
P06909	Complement factor H OS=Mus musculus GN=Cfh PE=1 SV=2	Cfh	P06909	0.318253642	0.785750244
P07309	Transthyretin OS=Mus musculus GN=Ttr PE=1 SV=1	Ttr	P07309	0.406988132	0.785750244
P07361	Alpha-1-acid glycoprotein 2 OS=Mus musculus GN=Orm2 PE=1 SV=1	Orm2	P07361	0.576540369	0.785750244
P07901	Heat shock protein HSP 90-alpha OS=Mus musculus GN=Hsp90aa1 PE=1 SV=4	Hsp90aa1	P07901	0.407301567	0.785750244
P08071	Lactotransferrin OS=Mus musculus GN=Ltf PE=1 SV=4	Ltf	P08071	0.361105943	0.785750244
P08730	Keratin, type I cytoskeletal 13 OS=Mus musculus GN=Krt13 PE=1 SV=2	Krt13	P08730	0.572406704	0.785750244
P09036	Serine protease inhibitor Kazal-type 3 OS=Mus musculus GN=Spink3 PE=1 SV=1	Spink3	P09036	0.502272273	0.785750244
P09411	Phosphoglycerate kinase 1 OS=Mus musculus GN=Pgk1 PE=1 SV=4	Pgk1	P09411	0.500624705	0.785750244
P09803	Cadherin-1 OS=Mus musculus GN=Cdh1 PE=1 SV=1	Cdh1	P09803	0.407301567	0.785750244
P10404	MLV-related proviral Env polyprotein OS=Mus musculus PE=1 SV=3	0	P10404	0.567907707	0.785750244
P10649	Glutathione S-transferase Mu 1 OS=Mus musculus GN=Gstm1 PE=1 SV=2	Gstm1	P10649	0.572406704	0.785750244
P11087	Collagen alpha-1(I) chain OS=Mus musculus GN=Col1a1 PE=1 SV=4	Col1a1	P11087	0.316675696	0.785750244
P11438	Lysosome-associated membrane glycoprotein 1 OS=Mus musculus GN=Lamp1 PE=1 SV=2	Lamp1	P11438	0.572378955	0.785750244
P11499	Heat shock protein HSP 90-beta OS=Mus musculus GN=Hsp90ab1 PE=1 SV=3	Hsp90ab1	P11499	0.572406704	0.785750244
P11588	Major urinary protein 1 OS=Mus musculus GN=Mup1 PE=1 SV=1	Mup1	P11588	0.534575948	0.785750244
P11672	Neutrophil gelatinase-associated lipocalin OS=Mus musculus GN=Lcn2 PE=1 SV=1	Lcn2	P11672	0.572406704	0.785750244
P11859	Angiotensinogen OS=Mus musculus GN=Agt PE=1 SV=1	Agt	P11859	0.369728789	0.785750244
P13595	Neural cell adhesion molecule 1 OS=Mus musculus GN=Ncam1 PE=1 SV=3	Ncam1	P13595	0.451588009	0.785750244
P13597	Intercellular adhesion molecule 1 OS=Mus musculus GN=Icam1 PE=1 SV=1	Icam1	P13597	0.402996876	0.785750244
P14106	Complement C1q subcomponent subunit B OS=Mus musculus GN=C1qb PE=1 SV=2	C1qb	P14106	0.530165854	0.785750244
P15209	BDNF/NT-3 growth factors receptor OS=Mus musculus GN=Ntrk2 PE=1 SV=1	Ntrk2	P15209	0.572406704	0.785750244
P15327	Bisphosphoglycerate mutase OS=Mus musculus GN=Bpgm PE=1 SV=2	Bpgm	P15327	0.306797497	0.785750244
P15379	CD44 antigen OS=Mus musculus GN=Cd44 PE=1 SV=3	Cd44	P15379	0.384904801	0.785750244
P16294	Coagulation factor IX OS=Mus musculus GN=F9 PE=2 SV=3	F9	P16294	0.48851174	0.785750244
P16675	Lysosomal protective protein OS=Mus musculus GN=Ctsa PE=1 SV=1	Ctsa	P16675	0.493955131	0.785750244
P17182	Alpha-enolase OS=Mus musculus GN=Eno1 PE=1 SV=3	Eno1	P17182	0.38339524	0.785750244
P18242	Cathepsin D OS=Mus musculus GN=Ctsd PE=1 SV=1	Ctsd	P18242	0.407301567	0.785750244
P18531	Ig heavy chain V region 3-6 OS=Mus musculus GN=Ighv3-6 PE=1 SV=1	Ighv3-6	P18531	0.407301567	0.785750244
P19157	Glutathione S-transferase P 1 OS=Mus musculus GN=Gstp1 PE=1 SV=2	Gstp1	P19157	0.564046383	0.785750244
P20029	78 kDa glucose-regulated protein OS=Mus musculus GN=Hspa5 PE=1 SV=3	Hspa5	P20029	0.373687233	0.785750244
P20152	Vimentin OS=Mus musculus GN=Vim PE=1 SV=3	Vim	P20152	0.572406704	0.785750244
P21180	Complement C2 OS=Mus musculus GN=C2 PE=1 SV=2	C2	P21180	0.496792928	0.785750244
P21956	Lactadherin OS=Mus musculus GN=Mfge8 PE=1 SV=3	Mfge8	P21956	0.407301567	0.785750244
P23492	Purine nucleoside phosphorylase OS=Mus musculus GN=Pnp PE=1 SV=2	Pnp	P23492	0.549657252	0.785750244
P24549	Retinal dehydrogenase 1 OS=Mus musculus GN=Aldh1a1 PE=1 SV=5	Aldh1a1	P24549	0.479107783	0.785750244
P27661	Histone H2AX OS=Mus musculus GN=H2afx PE=1 SV=2	H2afx	P27661	0.429421025	0.785750244

P27931	Interleukin-1 receptor type 2 OS=Mus musculus GN=Il1r2 PE=1 SV=1	Il1r2	P27931	0.564983023	0.785750244
P29699	Alpha-2-HS-glycoprotein OS=Mus musculus GN=Ahsg PE=1 SV=1	Ahsg	P29699	0.538598691	0.785750244
P29788	Vitronectin OS=Mus musculus GN=Vtn PE=1 SV=2	Vtn	P29788	0.466185774	0.785750244
P33175	Kinesin heavy chain isoform 5A OS=Mus musculus GN=Kif5a PE=1 SV=3	Kif5a	P33175	0.572406704	0.785750244
P33434	72 kDa type IV collagenase OS=Mus musculus GN=Mmp2 PE=1 SV=1	Mmp2	P33434	0.407301567	0.785750244
P33587	Vitamin K-dependent protein C OS=Mus musculus GN=Proc PE=1 SV=2	Proc	P33587	0.479107783	0.785750244
P34902	Cytokine receptor common subunit gamma OS=Mus musculus GN=Il2rg PE=2 SV=1	Il2rg	P34902	0.319986664	0.785750244
P34928	Apolipoprotein C-I OS=Mus musculus GN=Apoc1 PE=1 SV=1	Apoc1	P34928	0.3350243	0.785750244
P35505	Fumarylacetoacetase OS=Mus musculus GN=Fah PE=1 SV=2	Fah	P35505	0.564046383	0.785750244
P38647	Stress-70 protein, mitochondrial OS=Mus musculus GN=Hspa9 PE=1 SV=3	Hspa9	P38647	0.572406704	0.785750244
P40124	Adenylyl cyclase-associated protein 1 OS=Mus musculus GN=Cap1 PE=1 SV=4	Cap1	P40124	0.572406704	0.785750244
P40142	Transketolase OS=Mus musculus GN=Tkt PE=1 SV=1	Tkt	P40142	0.479107783	0.785750244
P43025	Tetranectin OS=Mus musculus GN=Clec3b PE=1 SV=2	Clec3b	P43025	0.397610577	0.785750244
P47879	Insulin-like growth factor-binding protein 4 OS=Mus musculus GN=Igfbp4 PE=1 SV=2	Igfbp4	P47879	0.361552871	0.785750244
P47968	Ribose-5-phosphate isomerase OS=Mus musculus GN=Rpia PE=1 SV=2	Rpia	P47968	0.572406704	0.785750244
P49182	Heparin cofactor 2 OS=Mus musculus GN=Serpind1 PE=1 SV=1	Serpind1	P49182	0.527338464	0.785750244
P52480-2	Isoform M1 of Pyruvate kinase PKM OS=Mus musculus GN=Pkm	Pkm	P52480-2	0.431579376	0.785750244
P56395	Cytochrome b5 OS=Mus musculus GN=Cyb5a PE=1 SV=2	Cyb5a	P56395	0.48851174	0.785750244
P61460	DEP domain-containing protein 5 OS=Mus musculus GN=Depdc5 PE=1 SV=2	Depdc5	P61460	0.407301567	0.785750244
P61939	Thyroxine-binding globulin OS=Mus musculus GN=Serpina7 PE=2 SV=1	Serpina7	P61939	0.514892477	0.785750244
P62774	Myotrophin OS=Mus musculus GN=Mtpn PE=1 SV=2	Mtpn	P62774	0.572406704	0.785750244
P62983	Ubiquitin-40S ribosomal protein S27a OS=Mus musculus GN=Rps27a PE=1 SV=2	Rps27a	P62983	0.407301567	0.785750244
P63101	14-3-3 protein zeta/delta OS=Mus musculus GN=Ywhaz PE=1 SV=1	Ywhaz	P63101	0.407301567	0.785750244
P68254	14-3-3 protein theta OS=Mus musculus GN=Ywhaq PE=1 SV=1	Ywhaq	P68254	0.572406704	0.785750244
P68372	Tubulin beta-4B chain OS=Mus musculus GN=Tubb4b PE=1 SV=1	Tubb4b	P68372	0.572406704	0.785750244
P70194	C-type lectin domain family 4 member F OS=Mus musculus GN=Clec4f PE=1 SV=1	Clec4f	P70194	0.572406704	0.785750244
P70375	Coagulation factor VII OS=Mus musculus GN=F7 PE=2 SV=1	F7	P70375	0.407301567	0.785750244
P70389	Insulin-like growth factor-binding protein complex acid labile subunit OS=Mus musculus GN=Igfals PE=1 SV=1	Igfals	P70389	0.482202614	0.785750244
P80316	T-complex protein 1 subunit epsilon OS=Mus musculus GN=Cct5 PE=1 SV=1	Cct5	P80316	0.479107783	0.785750244
P97298	Pigment epithelium-derived factor OS=Mus musculus GN=Serpinf1 PE=1 SV=2	Serpinf1	P97298	0.52367186	0.785750244
P97333	Neuropilin-1 OS=Mus musculus GN=Nrp1 PE=1 SV=2	Nrp1	P97333	0.534063422	0.785750244
P98064	Mannan-binding lectin serine protease 1 OS=Mus musculus GN=Masp1 PE=1 SV=2	Masp1	P98064	0.479107783	0.785750244
P98064-2	Isoform 2 of Mannan-binding lectin serine protease 1 OS=Mus musculus GN=Masp1	Masp1	P98064-2	0.48851174	0.785750244
P99026	Proteasome subunit beta type-4 OS=Mus musculus GN=Psmb4 PE=1 SV=1	Psmb4	P99026	0.310054976	0.785750244
Q00897	Alpha-1-antitrypsin 1-4 OS=Mus musculus GN=Serpina1d PE=1 SV=1	Serpina1d	Q00897	0.447153149	0.785750244
Q00898	Alpha-1-antitrypsin 1-5 OS=Mus musculus GN=Serpina1e PE=1 SV=1	Serpina1e	Q00898	0.320614419	0.785750244
Q01102	P-selectin OS=Mus musculus GN=Selp PE=1 SV=1	Selp	Q01102	0.572406704	0.785750244
Q01853	Transitional endoplasmic reticulum ATPase OS=Mus musculus GN=Vcp PE=1 SV=4	Vcp	Q01853	0.407301567	0.785750244
Q02257	Junction plakoglobin OS=Mus musculus GN=Jup PE=1 SV=3	Jup	Q02257	0.572406704	0.785750244
Q03311	Cholinesterase OS=Mus musculus GN=Bche PE=1 SV=2	Bche	Q03311	0.438551145	0.785750244
Q05020	Apolipoprotein C-II OS=Mus musculus GN=Apoc2 PE=2 SV=1	Apoc2	Q05020	0.463294311	0.785750244
Q07079	Insulin-like growth factor-binding protein 5 OS=Mus musculus GN=Igfbp5 PE=1 SV=1	Igfbp5	Q07079	0.407301567	0.785750244
Q08761	Vitamin K-dependent protein S OS=Mus musculus GN=Pros1 PE=2 SV=1	Pros1	Q08761	0.48851174	0.785750244
Q08879	Fibulin-1 OS=Mus musculus GN=Fbln1 PE=1 SV=2	Fbln1	Q08879	0.47435427	0.785750244
Q3TTY5	Keratin, type II cytoskeletal 2 epidermal OS=Mus musculus GN=Krt2 PE=1 SV=1	Krt2	Q3TTY5	0.450977792	0.785750244
Q3UPP8	Centrosomal protein of 63 kDa OS=Mus musculus GN=Cep63 PE=1 SV=2	Cep63	Q3UPP8	0.572406704	0.785750244
Q5M6W3	Clathrin heavy chain linker domain-containing protein 1 OS=Mus musculus GN=Clhc1 PE=2 SV=1	Clhc1	Q5M6W3	0.453790623	0.785750244
Q5RJH6-2	Isoform 2 of Protein SMG7 OS=Mus musculus GN=Smg7	Smg7	Q5RJH6-2	0.572406704	0.785750244
Q60590	Alpha-1-acid glycoprotein 1 OS=Mus musculus GN=Orm1 PE=1 SV=1	Orm1	Q60590	0.460962241	0.785750244
Q60692	Proteasome subunit beta type-6 OS=Mus musculus GN=Psmb6 PE=1 SV=3	Psmb6	Q60692	0.480207644	0.785750244
Q61043	Ninein OS=Mus musculus GN=Nin PE=1 SV=3	Nin	Q61043	0.572406704	0.785750244
Q61233	Plastin-2 OS=Mus musculus GN=Lcp1 PE=1 SV=4	Lcp1	Q61233	0.564046383	0.785750244
Q61268	Apolipoprotein C-IV OS=Mus musculus GN=Apoc4 PE=1 SV=1	Apoc4	Q61268	0.47319229	0.785750244
Q61598	Rab GDP dissociation inhibitor beta OS=Mus musculus GN=Gdi2 PE=1 SV=1	Gdi2	Q61598	0.572406704	0.785750244
Q61702	Inter-alpha-trypsin inhibitor heavy chain H1 OS=Mus musculus GN=Itih1 PE=1 SV=2	Itih1	Q61702	0.487569793	0.785750244
Q61704	Inter-alpha-trypsin inhibitor heavy chain H3 OS=Mus musculus GN=Itih3 PE=1 SV=3	Itih3	Q61704	0.577186908	0.785750244
Q61830	Macrophage mannose receptor 1 OS=Mus musculus GN=Mrc1 PE=1 SV=2	Mrc1	Q61830	0.407301567	0.785750244
Q62009	Periostin OS=Mus musculus GN=Postn PE=1 SV=2	Postn	Q62009	0.518883821	0.785750244
Q64726	Zinc-alpha-2-glycoprotein OS=Mus musculus GN=Azgp1 PE=1 SV=2	Azgp1	Q64726	0.334950549	0.785750244
Q6IME9	Keratin, type II cytoskeletal 72 OS=Mus musculus GN=Krt72 PE=3 SV=1	Krt72	Q6IME9	0.480746887	0.785750244
Q6P5H2	Nestin OS=Mus musculus GN=Nes PE=1 SV=1	Nes	Q6P5H2	0.572406704	0.785750244
Q6PFE3	DNA repair and recombination protein RAD54B OS=Mus musculus GN=Rad54b PE=2 SV=1	Rad54b	Q6PFE3	0.407301567	0.785750244
Q71KU9	Fibrinogen-like protein 1 OS=Mus musculus GN=Fgl1 PE=1 SV=2	Fgl1	Q71KU9	0.422602983	0.785750244
Q7TMD7	Desmoglein-4 OS=Mus musculus GN=Dsg4 PE=1 SV=1	Dsg4	Q7TMD7	0.572406704	0.785750244
Q7TSA6	Proline and serine-rich protein 3 OS=Mus musculus GN=Proser3 PE=1 SV=1	Proser3	Q7TSA6	0.407301567	0.785750244
Q8BH35	Complement component C8 beta chain OS=Mus musculus GN=C8b PE=1 SV=1	C8b	Q8BH35	0.419661946	0.785750244
Q8BH61	Coagulation factor XIII A chain OS=Mus musculus GN=F13a1 PE=1 SV=3	F13a1	Q8BH61	0.458997705	0.785750244
Q8BHC4	Dephospho-CoA kinase domain-containing protein OS=Mus musculus GN=Dcakd PE=1 SV=1	Dcakd	Q8BHC4	0.407301567	0.785750244
Q8BJA3	Homeobox-containing protein 1 OS=Mus musculus GN=Hmbox1 PE=1 SV=1	Hmbox1	Q8BJA3	0.572406704	0.785750244
Q8BQZ4	Ral GTPase-activating protein subunit beta OS=Mus musculus GN=Ralgapb PE=1 SV=2	Ralgapb	Q8BQZ4	0.572406704	0.785750244
Q8C255-2	Isoform 2 of Dipeptidase 2 OS=Mus musculus GN=Dpep2	Dpep2	Q8C255-2	0.572406704	0.785750244
Q8C5Q4	G-rich sequence factor 1 OS=Mus musculus GN=Grsf1 PE=1 SV=2	Grsf1	Q8C5Q4	0.572406704	0.785750244
Q8CG14	Complement C1s-A subcomponent OS=Mus musculus GN=C1sa PE=2 SV=2	C1sa	Q8CG14	0.48851174	0.785750244
Q8K0E8	Fibrinogen beta chain OS=Mus musculus GN=Fgb PE=1 SV=1	Fgb	Q8K0E8	0.387131253	0.785750244
Q8R016	Bleomycin hydrolase OS=Mus musculus GN=Blmh PE=1 SV=1	Blmh	Q8R016	0.407301567	0.785750244
Q8R081	Heterogeneous nuclear ribonucleoprotein L OS=Mus musculus GN=Hnrnpl PE=1 SV=2	Hnrnpl	Q8R081	0.572406704	0.785750244
Q8R2Y2	Cell surface glycoprotein MUC18 OS=Mus musculus GN=Mcam PE=1 SV=1	Mcam	Q8R2Y2	0.407301567	0.785750244
Q8VCG4	Complement component C8 gamma chain OS=Mus musculus GN=C8g PE=1 SV=1	C8g	Q8VCG4	0.570505289	0.785750244
Q91VV4	DENN domain-containing protein 2D OS=Mus musculus GN=Dennd2d PE=1 SV=1	Dennd2d	Q91VV4	0.572406704	0.785750244
Q91WP6	Serine protease inhibitor A3N OS=Mus musculus GN=Serpina3n PE=1 SV=1	Serpina3n	Q91WP6	0.315490167	0.785750244
Q91X17	Uromodulin OS=Mus musculus GN=Umod PE=1 SV=1	Umod	Q91X17	0.314253687	0.785750244
Q91X72	Hemopexin OS=Mus musculus GN=Hpx PE=1 SV=2	Hpx	Q91X72	0.566602781	0.785750244
Q91Z98	Chitinase-like protein 4 OS=Mus musculus GN=Chil4 PE=1 SV=2	Chil4	Q91Z98	0.407301567	0.785750244

Q923D2	Flavin reductase (NADPH) OS=Mus musculus GN=Blvrb PE=1 SV=3	Blvrb	Q923D2	0.577088894	0.785750244
Q99N48	Synaptotagmin-like protein 3 OS=Mus musculus GN=Sytl3 PE=1 SV=2	Sytl3	Q99N48	0.572406704	0.785750244
Q9CPU0	Lactoylglutathione lyase OS=Mus musculus GN=Glo1 PE=1 SV=3	Glo1	Q9CPU0	0.572406704	0.785750244
Q9CQ48	NudC domain-containing protein 2 OS=Mus musculus GN=Nudcd2 PE=1 SV=1	Nudcd2	Q9CQ48	0.407301567	0.785750244
Q9CQF9	Prenylcysteine oxidase OS=Mus musculus GN=Pcyox1 PE=1 SV=1	Pcyox1	Q9CQF9	0.37862344	0.785750244
Q9CQI6	Coactosin-like protein OS=Mus musculus GN=Cotl1 PE=1 SV=3	Cotl1	Q9CQI6	0.572406704	0.785750244
Q9CQW3	Vitamin K-dependent protein Z OS=Mus musculus GN=Proz PE=1 SV=1	Proz	Q9CQW3	0.369441216	0.785750244
Q9D1H9	Microfibril-associated glycoprotein 4 OS=Mus musculus GN=Mfap4 PE=1 SV=1	Mfap4	Q9D1H9	0.407301567	0.785750244
Q9D358	Low molecular weight phosphotyrosine protein phosphatase OS=Mus musculus GN=Acp1 PE=1 SV=3	Acp1	Q9D358	0.518415795	0.785750244
Q9DBD0	Inhibitor of carbonic anhydrase OS=Mus musculus GN=Ica PE=1 SV=1	Ica	Q9DBD0	0.477452388	0.785750244
Q9DBJ1	Phosphoglycerate mutase 1 OS=Mus musculus GN=Pgam1 PE=1 SV=3	Pgam1	Q9DBJ1	0.403507283	0.785750244
Q9DC11	Plexin domain-containing protein 2 OS=Mus musculus GN=Plxdc2 PE=1 SV=1	Plxdc2	Q9DC11	0.564046383	0.785750244
Q9DCD0	6-phosphogluconate dehydrogenase, decarboxylating OS=Mus musculus GN=Pgd PE=1 SV=3	Pgd	Q9DCD0	0.572406704	0.785750244
Q9EQY0	Serine/threonine-protein kinase/endoribonuclease IRE1 OS=Mus musculus GN=Ern1 PE=1 SV=1	Ern1	Q9EQY0	0.407301567	0.785750244
Q9ESB3	Histidine-rich glycoprotein OS=Mus musculus GN=Hrg PE=1 SV=2	Hrg	Q9ESB3	0.344654081	0.785750244
Q9ESY9	Gamma-interferon-inducible lysosomal thiol reductase OS=Mus musculus GN=Ifi30 PE=1 SV=3	Ifi30	Q9ESY9	0.343863946	0.785750244
Q9JIL4	Na(+)/H(+) exchange regulatory cofactor NHE-RF3 OS=Mus musculus GN=Pdzk1 PE=1 SV=1	Pdzk1	Q9JIL4	0.407301567	0.785750244
Q9R097	Kunitz-type protease inhibitor 1 OS=Mus musculus GN=Spint1 PE=1 SV=2	Spint1	Q9R097	0.352438178	0.785750244
Q9R0Y5-2	Isoform 2 of Adenylate kinase isoenzyme 1 OS=Mus musculus GN=Ak1	Ak1	Q9R0Y5-2	0.572406704	0.785750244
Q9R1E6-2	Isoform 2 of Ectonucleotide pyrophosphatase/phosphodiesterase family member 2 OS=Mus musculus GN=Enpp2	Enpp2	Q9R1E6-2	0.407301567	0.785750244
Q9R1P3	Proteasome subunit beta type-2 OS=Mus musculus GN=Psmb2 PE=1 SV=1	Psmb2	Q9R1P3	0.534047849	0.785750244
Q9WVA4	Transgelin-2 OS=Mus musculus GN=Tagln2 PE=1 SV=4	Tagln2	Q9WVA4	0.572406704	0.785750244
Q9Z0L8	Gamma-glutamyl hydrolase OS=Mus musculus GN=Ggh PE=1 SV=2	Ggh	Q9Z0L8	0.407301567	0.785750244
Q9Z121	C-C motif chemokine 8 OS=Mus musculus GN=Ccl8 PE=3 SV=1	Ccl8	Q9Z121	0.407301567	0.785750244
Q9Z2U1	Proteasome subunit alpha type-5 OS=Mus musculus GN=Psma5 PE=1 SV=1	Psma5	Q9Z2U1	0.363539824	0.785750244
Q9Z331	Keratin, type II cytoskeletal 6B OS=Mus musculus GN=Krt6b PE=1 SV=3	Krt6b	Q9Z331	0.564046383	0.785750244
P18337	L-selectin OS=Mus musculus GN=Sell PE=1 SV=1	Sell	P18337	0.578856306	0.785821689
P97399	Dentin sialophosphoprotein OS=Mus musculus GN=Dspp PE=1 SV=2	Dspp	P97399	0.586150683	0.791145646
Q2VIS4	Filaggrin-2 OS=Mus musculus GN=Flg2 PE=1 SV=2	Flg2	Q2VIS4	0.587661683	0.791145646
Q78ZA7	Nucleosome assembly protein 1-like 4 OS=Mus musculus GN=Nap1l4 PE=1 SV=1	Nap1l4	Q78ZA7	0.584573424	0.791145646
P11680	Properdin OS=Mus musculus GN=Cfp PE=2 SV=2	Cfp	P11680	0.592120816	0.792756795
Q91Y97	Fructose-bisphosphate aldolase B OS=Mus musculus GN=Aldob PE=1 SV=3	Aldob	Q91Y97	0.592111392	0.792756795
P16858	Glyceraldehyde-3-phosphate dehydrogenase OS=Mus musculus GN=Gapdh PE=1 SV=2	Gapdh	P16858	0.598337338	0.798432384
Q3U0B3	Dehydrogenase/reductase SDR family member 11 OS=Mus musculus GN=Dhrs11 PE=1 SV=1	Dhrs11	Q3U0B3	0.599645721	0.798432384
P01027	Complement C3 OS=Mus musculus GN=C3 PE=1 SV=3	C3	P01027	0.605347886	0.802122028
P32261	Antithrombin-III OS=Mus musculus GN=Serpinc1 PE=1 SV=1	Serpinc1	P32261	0.605717663	0.802122028
P05064	Fructose-bisphosphate aldolase A OS=Mus musculus GN=Aldoa PE=1 SV=2	Aldoa	P05064	0.615215038	0.812485077
E9Q414	Apolipoprotein B-100 OS=Mus musculus GN=Apob PE=1 SV=1	Apob	E9Q414	0.620655727	0.815239684
P06684	Complement C5 OS=Mus musculus GN=C5 PE=1 SV=2	C5	P06684	0.619435844	0.815239684
P62897	Cytochrome c, somatic OS=Mus musculus GN=Cycs PE=1 SV=2	Cycs	P62897	0.630542557	0.825993754
Q9Z126	Platelet factor 4 OS=Mus musculus GN=Pf4 PE=1 SV=1	Pf4	Q9Z126	0.632382903	0.826177664
O35930	Platelet glycoprotein Ib alpha chain OS=Mus musculus GN=Gp1ba PE=1 SV=2	Gp1ba	O35930	0.685520295	0.829174847
O55234	Proteasome subunit beta type-5 OS=Mus musculus GN=Psmb5 PE=1 SV=3	Psmb5	O55234	0.650082515	0.829174847
O70250	Phosphoglycerate mutase 2 OS=Mus musculus GN=Pgam2 PE=1 SV=3	Pgam2	O70250	0.677619252	0.829174847
P01639	Ig kappa chain V-V region MOPC 41 OS=Mus musculus GN=Gm5571 PE=1 SV=1	Gm5571	P01639	0.689953419	0.829174847
P03953	Complement factor D OS=Mus musculus GN=Cfd PE=1 SV=1	Cfd	P03953	0.688264784	0.829174847
P06745	Glucose-6-phosphate isomerase OS=Mus musculus GN=Gpi PE=1 SV=4	Gpi	P06745	0.689953419	0.829174847
P09470	Angiotensin-converting enzyme OS=Mus musculus GN=Ace PE=1 SV=3	Ace	P09470	0.645725598	0.829174847
P09813	Apolipoprotein A-II OS=Mus musculus GN=Apoa2 PE=1 SV=2	Apoa2	P09813	0.662923554	0.829174847
P11590	Major urinary protein 4 OS=Mus musculus GN=Mup4 PE=1 SV=1	Mup4	P11590	0.650585637	0.829174847
P11591	Major urinary protein 5 OS=Mus musculus GN=Mup5 PE=2 SV=1	Mup5	P11591	0.689953419	0.829174847
P17742	Peptidyl-prolyl cis-trans isomerase A OS=Mus musculus GN=Ppia PE=1 SV=2	Ppia	P17742	0.684244796	0.829174847
P23953	Carboxylesterase 1C OS=Mus musculus GN=Ces1c PE=1 SV=4	Ces1c	P23953	0.671090803	0.829174847
P35230	Regenerating islet-derived protein 3-beta OS=Mus musculus GN=Reg3b PE=1 SV=1	Reg3b	P35230	0.678327239	0.829174847
P51437	Cathelin-related antimicrobial peptide OS=Mus musculus GN=Camp PE=1 SV=1	Camp	P51437	0.690979039	0.829174847
P51910	Apolipoprotein D OS=Mus musculus GN=Apod PE=1 SV=1	Apod	P51910	0.653286094	0.829174847
P70195	Proteasome subunit beta type-7 OS=Mus musculus GN=Psmb7 PE=1 SV=1	Psmb7	P70195	0.679003775	0.829174847
P70663	SPARC-like protein 1 OS=Mus musculus GN=Sparcl1 PE=1 SV=3	Sparcl1	P70663	0.663024415	0.829174847
Q02105	Complement C1q subcomponent subunit C OS=Mus musculus GN=C1qc PE=1 SV=2	C1qc	Q02105	0.64051249	0.829174847
Q5I2A0	Serine protease inhibitor A3G OS=Mus musculus GN=Serpina3g PE=1 SV=2	Serpina3g	Q5I2A0	0.689953419	0.829174847
Q61129	Complement factor I OS=Mus musculus GN=Cfi PE=1 SV=3	Cfi	Q61129	0.671805395	0.829174847
Q62351	Transferrin receptor protein 1 OS=Mus musculus GN=Tfrc PE=1 SV=1	Tfrc	Q62351	0.679003775	0.829174847
Q64323	N-acetylglucosaminyl-phosphatidylinositol biosynthetic protein OS=Mus musculus GN=Piga PE=2 SV=1	Piga	Q64323	0.671618033	0.829174847
Q64522	Histone H2A type 2-B OS=Mus musculus GN=Hist2h2ab PE=1 SV=3	Hist2h2ab	Q64522	0.666917429	0.829174847
Q80XI7	Vomeromodulin OS=Mus musculus PE=2 SV=1	0	Q80XI7	0.689953419	0.829174847
Q8BPB5	EGF-containing fibulin-like extracellular matrix protein 1 OS=Mus musculus GN=Efemp1 PE=1 SV=1	Efemp1	Q8BPB5	0.638914102	0.829174847
Q8CIF4	Biotinidase OS=Mus musculus GN=Btd PE=1 SV=2	Btd	Q8CIF4	0.636417577	0.829174847
Q8K1I3	Secreted phosphoprotein 24 OS=Mus musculus GN=Spp2 PE=1 SV=2	Spp2	Q8K1I3	0.6580312	0.829174847
Q8VED5	Keratin, type II cytoskeletal 79 OS=Mus musculus GN=Krt79 PE=1 SV=2	Krt79	Q8VED5	0.674135262	0.829174847
Q9DAU7	WAP four-disulfide core domain protein 2 OS=Mus musculus GN=Wfdc2 PE=1 SV=1	Wfdc2	Q9DAU7	0.689953419	0.829174847
Q9EQH2	Endoplasmic reticulum aminopeptidase 1 OS=Mus musculus GN=Erap1 PE=1 SV=2	Erap1	Q9EQH2	0.679003775	0.829174847
Q9ET66	Peptidase inhibitor 16 OS=Mus musculus GN=Pi16 PE=2 SV=1	Pi16	Q9ET66	0.663047618	0.829174847
Q9JJH1	Ribonuclease 4 OS=Mus musculus GN=Rnase4 PE=1 SV=1	Rnase4	Q9JJH1	0.678699769	0.829174847
Q9Z1R3	Apolipoprotein M OS=Mus musculus GN=Apom PE=1 SV=1	Apom	Q9Z1R3	0.669449756	0.829174847
O08997	Copper transport protein ATOX1 OS=Mus musculus GN=Atox1 PE=1 SV=1	Atox1	O08997	0.699136705	0.835726331
P47876	Insulin-like growth factor-binding protein 1 OS=Mus musculus GN=Igfbp1 PE=2 SV=2	Igfbp1	P47876	0.703202216	0.835726331
Q8K0D2	Hyaluronan-binding protein 2 OS=Mus musculus GN=Habp2 PE=1 SV=2	Habp2	Q8K0D2	0.700609382	0.835726331
Q9JJN5	Carboxypeptidase N catalytic chain OS=Mus musculus GN=Cpn1 PE=1 SV=1	Cpn1	Q9JJN5	0.703317015	0.835726331
P13020	Gelsolin OS=Mus musculus GN=Gsn PE=1 SV=3	Gsn	P13020	0.708095758	0.838673129
P13634	Carbonic anhydrase 1 OS=Mus musculus GN=Ca1 PE=1 SV=4	Ca1	P13634	0.710872911	0.838673129
Q00724	Retinol-binding protein 4 OS=Mus musculus GN=Rbp4 PE=1 SV=2	Rbp4	Q00724	0.711119881	0.838673129
Q8CG16	Complement C1r-A subcomponent OS=Mus musculus GN=C1ra PE=1 SV=1	C1ra	Q8CG16	0.716150923	0.838673129
Q9JHJ8-2	Isoform 2 of ICOS ligand OS=Mus musculus GN=Icoslg	Icoslg	Q9JHJ8-2	0.715086473	0.838673129

Q9WVJ3	Carboxypeptidase Q OS=Mus musculus GN=Cpq PE=1 SV=1	Cpq	Q9WVJ3	0.714923198	0.838673129
Q9QWK4	CD5 antigen-like OS=Mus musculus GN=Cd5l PE=1 SV=3	Cd5l	Q9QWK4	0.719064021	0.84006037
P70269	Cathepsin E OS=Mus musculus GN=Ctse PE=1 SV=2	Ctse	P70269	0.723626091	0.843362782
Q9R1P1	Proteasome subunit beta type-3 OS=Mus musculus GN=Psmb3 PE=1 SV=1	Psmb3	Q9R1P1	0.727608354	0.845975264
P41317	Mannose-binding protein C OS=Mus musculus GN=Mbl2 PE=1 SV=2	Mbl2	P41317	0.734857444	0.851667417
Q01339	Beta-2-glycoprotein 1 OS=Mus musculus GN=Apoh PE=1 SV=1	Apoh	Q01339	0.739513683	0.851667417
Q8R121	Protein Z-dependent protease inhibitor OS=Mus musculus GN=Serpina10 PE=1 SV=1	Serpina10	Q8R121	0.736758846	0.851667417
Q93092	Transaldolase OS=Mus musculus GN=Taldo1 PE=1 SV=2	Taldo1	Q93092	0.739444467	0.851667417
P05367	Serum amyloid A-2 protein OS=Mus musculus GN=Saa2 PE=1 SV=1	Saa2	P05367	0.750018516	0.859691034
P47877	Insulin-like growth factor-binding protein 2 OS=Mus musculus GN=Igfbp2 PE=2 SV=2	Igfbp2	P47877	0.748837854	0.859691034
O08677-2	Isoform LMW of Kininogen-1 OS=Mus musculus GN=Kng1	Kng1	O08677-2	0.762780971	0.872262475
P97321	Prolyl endopeptidase FAP OS=Mus musculus GN=Fap PE=1 SV=1	Fap	P97321	0.771693609	0.88038285
Q01149	Collagen alpha-2(I) chain OS=Mus musculus GN=Col1a2 PE=1 SV=2	Col1a2	Q01149	0.774425036	0.881429901
P07743	BPI fold-containing family A member 2 OS=Mus musculus GN=Bpifa2 PE=1 SV=1	Bpifa2	P07743	0.778081464	0.881462917
Q8BHC0	Lymphatic vessel endothelial hyaluronic acid receptor 1 OS=Mus musculus GN=Lyve1 PE=1 SV=1	Lyve1	Q8BHC0	0.777298698	0.881462917
P16301	Phosphatidylcholine-sterol acyltransferase OS=Mus musculus GN=Lcat PE=1 SV=2	Lcat	P16301	0.787106314	0.889613183
P55065	Phospholipid transfer protein OS=Mus musculus GN=Pltp PE=1 SV=1	Pltp	P55065	0.796867596	0.895707119
Q05909	Receptor-type tyrosine-protein phosphatase gamma OS=Mus musculus GN=Ptprg PE=1 SV=1	Ptprg	Q05909	0.796954508	0.895707119
Q91X79	Chymotrypsin-like elastase family member 1 OS=Mus musculus GN=Cela1 PE=1 SV=1	Cela1	Q91X79	0.798027125	0.895707119
O55042	Alpha-synuclein OS=Mus musculus GN=Snca PE=1 SV=2	Snca	O55042	0.801205137	0.896165199
P11276	Fibronectin OS=Mus musculus GN=Fn1 PE=1 SV=4	Fn1	P11276	0.804784809	0.896165199
Q64442	Sorbitol dehydrogenase OS=Mus musculus GN=Sord PE=1 SV=3	Sord	Q64442	0.803625112	0.896165199
Q9Z2U0	Proteasome subunit alpha type-7 OS=Mus musculus GN=Psma7 PE=1 SV=1	Psma7	Q9Z2U0	0.805811094	0.896165199
Q60994	Adiponectin OS=Mus musculus GN=Adipoq PE=1 SV=2	Adipoq	Q60994	0.808607149	0.897221631
O35698	RNA-binding motif protein, Y chromosome, family 1 member A1 OS=Mus musculus GN=Rbmy1a1 PE=1 SV=2	Rbmy1a1	O35698	0.821981524	0.899736533
O70570	Polymeric immunoglobulin receptor OS=Mus musculus GN=Pigr PE=1 SV=1	Pigr	O70570	0.817800735	0.899736533
P28666	Murinoglobulin-2 OS=Mus musculus GN=Mug2 PE=1 SV=2	Mug2	P28666	0.817110199	0.899736533
P62962	Profilin-1 OS=Mus musculus GN=Pfn1 PE=1 SV=2	Pfn1	P62962	0.821981524	0.899736533
Q60805	Tyrosine-protein kinase Mer OS=Mus musculus GN=Mertk PE=1 SV=1	Mertk	Q60805	0.819424347	0.899736533
Q6GQT1	Alpha-2-macroglobulin-P OS=Mus musculus GN=A2mp PE=2 SV=2	A2mp	Q6GQT1	0.8149723	0.899736533
P08905	Lysozyme C-2 OS=Mus musculus GN=Lyz2 PE=1 SV=2	Lyz2	P08905	0.828378486	0.904700998
P52430	Serum paraoxonase/arylesterase 1 OS=Mus musculus GN=Pon1 PE=1 SV=2	Pon1	P52430	0.838598615	0.911683837
Q61730	Interleukin-1 receptor accessory protein OS=Mus musculus GN=Il1rap PE=1 SV=1	Il1rap	Q61730	0.840399915	0.911683837
Q9R0P3	S-formylglutathione hydrolase OS=Mus musculus GN=Esd PE=1 SV=1	Esd	Q9R0P3	0.838315667	0.911683837
P26928	Hepatocyte growth factor-like protein OS=Mus musculus GN=Mst1 PE=2 SV=2	Mst1	P26928	0.842616043	0.912052109
P11589	Major urinary protein 2 OS=Mus musculus GN=Mup2 PE=1 SV=1	Mup2	P11589	0.849300167	0.915210379
P21614	Vitamin D-binding protein OS=Mus musculus GN=Gc PE=1 SV=2	Gc	P21614	0.848628282	0.915210379
Q06770	Corticosteroid-binding globulin OS=Mus musculus GN=Serpina6 PE=1 SV=1	Serpina6	Q06770	0.85599042	0.918347338
Q61838	Alpha-2-macroglobulin OS=Mus musculus GN=A2m PE=1 SV=3	A2m	Q61838	0.854597394	0.918347338
Q61508	Extracellular matrix protein 1 OS=Mus musculus GN=Ecm1 PE=1 SV=2	Ecm1	Q61508	0.859863305	0.92047041
P31532	Serum amyloid A-4 protein OS=Mus musculus GN=Saa4 PE=1 SV=2	Saa4	P31532	0.866720893	0.92441303
Q921I1	Serotransferrin OS=Mus musculus GN=Tf PE=1 SV=1	Tf	Q921I1	0.867350497	0.92441303
Q8VCM7	Fibrinogen gamma chain OS=Mus musculus GN=Fgg PE=1 SV=1	Fgg	Q8VCM7	0.872601842	0.927974825
Q07968	Coagulation factor XIII B chain OS=Mus musculus GN=F13b PE=1 SV=2	F13b	Q07968	0.875522245	0.929047622
P47878	Insulin-like growth factor-binding protein 3 OS=Mus musculus GN=Igfbp3 PE=2 SV=2	Igfbp3	P47878	0.881821262	0.933693101
Q9QXC1	Fetuin-B OS=Mus musculus GN=Fetub PE=1 SV=1	Fetub	Q9QXC1	0.885446398	0.935493369
P05017-2	Isoform IGF-IB of Insulin-like growth factor I OS=Mus musculus GN=Igf1	Igf1	P05017-2	0.893252703	0.939655441
P12246	Serum amyloid P-component OS=Mus musculus GN=Apcs PE=1 SV=2	Apcs	P12246	0.892533908	0.939655441
Q61781	Keratin, type I cytoskeletal 14 OS=Mus musculus GN=Krt14 PE=1 SV=2	Krt14	Q61781	0.895987407	0.940496501
P01898	H-2 class I histocompatibility antigen, Q10 alpha chain OS=Mus musculus GN=H2-Q10 PE=1 SV=3	H2-Q10	P01898	0.907001919	0.95000632
P17047-2	Isoform LAMP-2B of Lysosome-associated membrane glycoprotein 2 OS=Mus musculus GN=Lamp2	Lamp2	P17047-2	0.910302403	0.951412834
P08607	C4b-binding protein OS=Mus musculus GN=C4bpa PE=1 SV=3	C4bpa	P08607	0.916306757	0.952932349
P14847	C-reactive protein OS=Mus musculus GN=Crp PE=1 SV=2	Crp	P14847	0.919027888	0.952932349
Q80YC5	Coagulation factor XII OS=Mus musculus GN=F12 PE=1 SV=2	F12	Q80YC5	0.919599324	0.952932349
Q9R1P4	Proteasome subunit alpha type-1 OS=Mus musculus GN=Psma1 PE=1 SV=1	Psma1	Q9R1P4	0.916930948	0.952932349
P70296	Phosphatidylethanolamine-binding protein 1 OS=Mus musculus GN=Pebp1 PE=1 SV=3	Pebp1	P70296	0.923461715	0.95489871
E9PV24	Fibrinogen alpha chain OS=Mus musculus GN=Fga PE=1 SV=1	Fga	E9PV24	0.93609307	0.965904951
Q8VCC2	Liver carboxylesterase 1 OS=Mus musculus GN=Ces1 PE=1 SV=1	Ces1	Q8VCC2	0.93944764	0.967312612
P20918	Plasminogen OS=Mus musculus GN=Plg PE=1 SV=3	Plg	P20918	0.945393034	0.971376352
Q03734	Serine protease inhibitor A3M OS=Mus musculus GN=Serpina3m PE=1 SV=2	Serpina3m	Q03734	0.948029452	0.972030198
P22599	Alpha-1-antitrypsin 1-2 OS=Mus musculus GN=Serpina1b PE=1 SV=2	Serpina1b	P22599	0.955697114	0.977829048
Q8BK48	Pyrethroid hydrolase Ces2e OS=Mus musculus GN=Ces2e PE=1 SV=1	Ces2e	Q8BK48	0.963187361	0.979307652
Q8VCS0	N-acetylmuramoyl-L-alanine amidase OS=Mus musculus GN=Pglyrp2 PE=1 SV=1	Pglyrp2	Q8VCS0	0.961479675	0.979307652
Q9R1P0	Proteasome subunit alpha type-4 OS=Mus musculus GN=Psma4 PE=1 SV=1	Psma4	Q9R1P0	0.962583132	0.979307652
P35700	Peroxiredoxin-1 OS=Mus musculus GN=Prdx1 PE=1 SV=1	Prdx1	P35700	0.965754422	0.979867743
P01844	Ig lambda-2 chain C region OS=Mus musculus GN=Iglc2 PE=1 SV=1	Iglc2	P01844	0.97179806	0.981899911
P19221	Prothrombin OS=Mus musculus GN=F2 PE=1 SV=1	F2	P19221	0.97178367	0.981899911
P02535	Keratin, type I cytoskeletal 10 OS=Mus musculus GN=Krt10 PE=1 SV=3	Krt10	P02535	0.98613357	0.991543347
P33622	Apolipoprotein C-III OS=Mus musculus GN=Apoc3 PE=1 SV=2	Apoc3	P33622	0.987462922	0.991543347
Q91WP0	Mannan-binding lectin serine protease 2 OS=Mus musculus GN=Masp2 PE=1 SV=1	Masp2	Q91WP0	0.984490513	0.991543347
P07724	Serum albumin OS=Mus musculus GN=Alb PE=1 SV=3	Alb	P07724	0.991680749	0.991680749
Q61805	Lipopolysaccharide-binding protein OS=Mus musculus GN=Lbp PE=1 SV=2	Lbp	Q61805	0.990604536	0.991680749

Supplementary Table 3: Protein abundances for untargeted proteomics analysis. Descriptions, Gene names, Uniprot accesions ID, p-values, and q-values are listed.
